# Cellular senescence in multiple sclerosis: hallmarks, immunosenescence, and therapeutic implications

**DOI:** 10.3389/fimmu.2026.1944657

**Published:** 2026-10-07

**Authors:** Ala’ Yousef, John M. Seubert, Christopher Power, Nabeela Nathoo

**Affiliations:** 1 Department of Medicine (Neurology), Faculty of Medicine and Dentistry, University of Alberta, Edmonton, AB, Canada; 2 Multiple Sclerosis Centre, University of Alberta, Edmonton, AB, Canada; 3 Faculty of Pharmacy and Pharmaceutical Sciences, University of Alberta, Edmonton, AB, Canada; 4 Department of Pharmacology, Faculty of Medicine and Dentistry, University of Alberta, Edmonton, AB, Canada

**Keywords:** aging, brain atrophy, cytomegalovirus (CMV), Epstein-Barr virus, immunosenescence, multiple sclerosis, senotherapeutics, telomere

## Abstract

Multiple sclerosis (MS) is an immune-mediated neurodegenerative disease where chronological aging predicts the conversion to progressive MS (PMS), a phase defined by compartmentalized inflammation and accelerated neurodegeneration. While current disease modifying therapies (DMTs) are effective for managing the inflammatory aspect of MS, treatments remain inadequate. It is imperative to understand the biology of aging including an important hallmark of aging, cellular senescence. Cellular senescence is a state of cell cycle arrest where cells remain in their senescent state and produce a distinctive senescence-associated secretory phenotype (SASP), characterized by the release of pro-inflammatory cytokines, chemokines, and growth factors. Growing experimental and clinical evidence suggests that the accumulation of senescent cells and SASP secretion perpetuates a pathological feedback loop linking age-related processes with accelerated neurodegeneration observed in PMS. Whether senescence drives PMS or accompanies it remains unresolved. In this Review, we outline the biology of aging, senescence pathway markers, and SASP components. We evaluate studies of cellular senescence and SASP markers in MS, including immunosenescence, T- and B-cell changes; the contributions of Epstein-Barr virus, cytomegalovirus, and herpes simplex virus-1; and the relationship between senescence burden, brain atrophy, and DMT response. Lastly, we outline the available senotherapeutic approaches aimed at mitigating MS and other neurodegenerative diseases.

## 1 Introduction

Multiple sclerosis (MS) is a chronic, progressive neurological disease with hallmark features including inflammation, demyelination, and neurodegeneration. The average age of MS onset typically falls between 20 and 40 years (1) with a disease duration of decades. More than 80% of people initially present with relapsing MS (RMS) (2), characterized by episodes of relapses with acute neurological dysfunction, followed by partial or full recovery. In the treatment era, approximately one in five people with RMS develop secondary progressive MS (SPMS), characterized by a shift from acute relapses to steady disability accumulation, a median of approximately 17 years after MS onset (3). A subset of people present with progressive disease from onset, referred to as primary progressive MS (PPMS) (2). Despite sharing the same chronological age, it is still unknown why some people progress and others do not, or why some people progress at a faster rate than others do. It is notable that the older the chronological age of MS onset, the faster the development of the progressive phase (4). People with late-onset MS (LOMS; greater than age 50 at MS onset) reach higher levels of irreversible disability sooner than those with pediatric-onset MS (POMS) (5). The opposite holds true for POMS, wherein progression takes longer to emerge, around 30 years from first symptoms, emphasizing that youthful age might delay the progressive phase. These clinical observations support the fact that age is a significant factor for the development of progression in MS (4, 6).

Chronological age represents the number of years a person has lived. It is a fixed concept determined by the calendar. Biological age indicates systemic functional decline and the cumulative molecular damage experienced over time with decreased physiological reserve to handle stress (7). Biological age can be higher or lower than chronological age in a given individual. Differences in the pace of biological aging among individuals may contribute to the clinical heterogeneity observed in persons with MS (pwMS) (8). Several theories have been proposed regarding the pathophysiology of biological aging, but there are still challenges in identifying a single biomarker or collection of biomarkers that reliably predicts the pace of aging. Nevertheless, prospective biomarkers that have been generated from the hallmarks of aging (9, 10) are starting to provide new insights on disease outcomes and progression in MS (11). These include, but are not limited to, genomic instability, stem cell exhaustion, chronic inflammation, mitochondrial dysfunction, and cellular senescence, all of which reflect the dysregulation of interconnected cellular functions (9, 10). Cellular senescence is defined as a state of cell cycle arrest in which cells permanently stop dividing while remaining metabolically active (12). In normal physiological conditions, cellular senescence contributes to embryonic development, wound healing and tumor suppression (13, 14). The purpose of this arrest is to limit the proliferation of damaged cells, to facilitate the elimination of accumulated harmful factors, and to prevent potential malignant transformation. However, problems arise with the long-term accumulation of senescent cells in various tissues through the release of proinflammatory factors, negatively affecting tissue environment and rendering them more susceptible to stress. There is a strong correlation between senescent cell accumulation and age-related diseases such as cardiovascular, pulmonary, kidney and neurodegenerative diseases (15–18).

Given the growing understanding of biological age in MS, in this Review, we outline senescence pathway markers and immunosenescence in the context of MS. We also evaluate senotherapeutic approaches used in MS and other neurodegenerative diseases.

## 2 Cellular senescence

In 1961, Hayflick and Moorhead discovered replicative senescence, observed as irreversible cell cycle arrest in cultured human fibroblasts after a finite number of divisions (19). Later, telomeres in human fibroblasts and vessels were reported to shorten with each cell division and have an effect on normal aging (20, 21). Telomeres are located at the ends of chromosomes and composed of repetitive nucleotides of TTAGGG (22). Their main function is to protect genetic information from damage during DNA replication and cell division (22). While central nervous system (CNS) mitotic activity remains generally low in adulthood, continuous cellular turnover leads to a gradual depletion of telomere length, ultimately resulting in short and dysfunctional telomeres (23). Telomere shortening, cellular senescence, and aging are all interconnected (24). Human data have demonstrated that telomere shortening is age-dependent and can be accelerated by genetic, environmental and lifestyle factors, including gut dysbiosis, smoking, chronic stress, and obesity (25, 26). However, several studies show that cellular senescence extends beyond telomere shortening and replicative senescence. For example, oxidative stress and chronic inflammation cause telomere damage independent of telomere length, which can trigger cellular senescence in various tissues, including the brain (27). Accordingly, cellular senescence encompasses three main types (1): developmental senescence which occurs during normal embryonic development (2); replicative senescence that is age-related, developing primarily as a consequence of telomere shortening (3); stress-induced premature senescence, which is triggered by oxidative or DNA damage (28–30). This is important as replicative senescence is typically not prominent at a younger age, but can be induced prematurely by increased reactive oxidative species, sustained chronic inflammation and immune activation. This is relevant to MS, where continuous activation of immune cells eventually leads to a senescent stage with reduced proliferative capacity and effector function (discussed in detail in Section 4: Immunosenescence in MS). Moreover, the severity and duration of cellular stress influence cell fate (31). Severe and acute stress activates regulated cell death pathways such as apoptosis, necroptosis, pyroptosis, and ferroptosis (32–35), whereas chronic continuous stress promotes cellular senescence, allowing cells to persist in their metabolically active state. Senescence and regulated cellular death pathways are interconnected (36). Through the secretion of SASP components, senescent cells activate cellular death pathways in adjacent cells while sustaining chronic inflammation and senescent microenvironment (31). This ability to alter tissue microenvironment distinguishes senescence from other regulated cell death pathways and highlights its importance in MS progression (37, 38).

### 2.1 Markers of cellular senescence

Senescent cells stay in their stable and growth-arrested state (12). The cell cycle arrest program is regulated through multiple molecular mechanisms. The main pathways involved in regulating cell cycle progression are p53/p21 and p16/Rb, which inhibit the cyclin-dependent kinases (CDKs) and block the activity of retinoblastoma (Rb) (39). Shorter telomeres and DNA damage responses activate the p53 pathway, a central regulator of cell-cycle control that is tightly regulated by post-translational modifications such as phosphorylation, acetylation, and ubiquitination (40, 41). Stabilization of p53 is further enhanced by phosphorylation through DNA damage response kinases, including ataxia-telangiectasia mutated (ATM) and ataxia-telangiectasia and Rad3-related (ATR), which increase p53 activity and protect it from degradation. This subsequently induces the expression of p21, a CDK inhibitor that primarily targets CDK2 (42). In parallel with continuous stress, the cell directly activates p16, which inhibits CDK4 and CDK6, preventing Rb phosphorylation and maintaining Rb in its active state (43). Both p53/p21 and p16/Rb pathways result in hypophosphorylation of Rb, arresting the cell cycle at the G1/S phase and maintaining the senescent state (**Figure 1**) (44).

**Figure 1:**
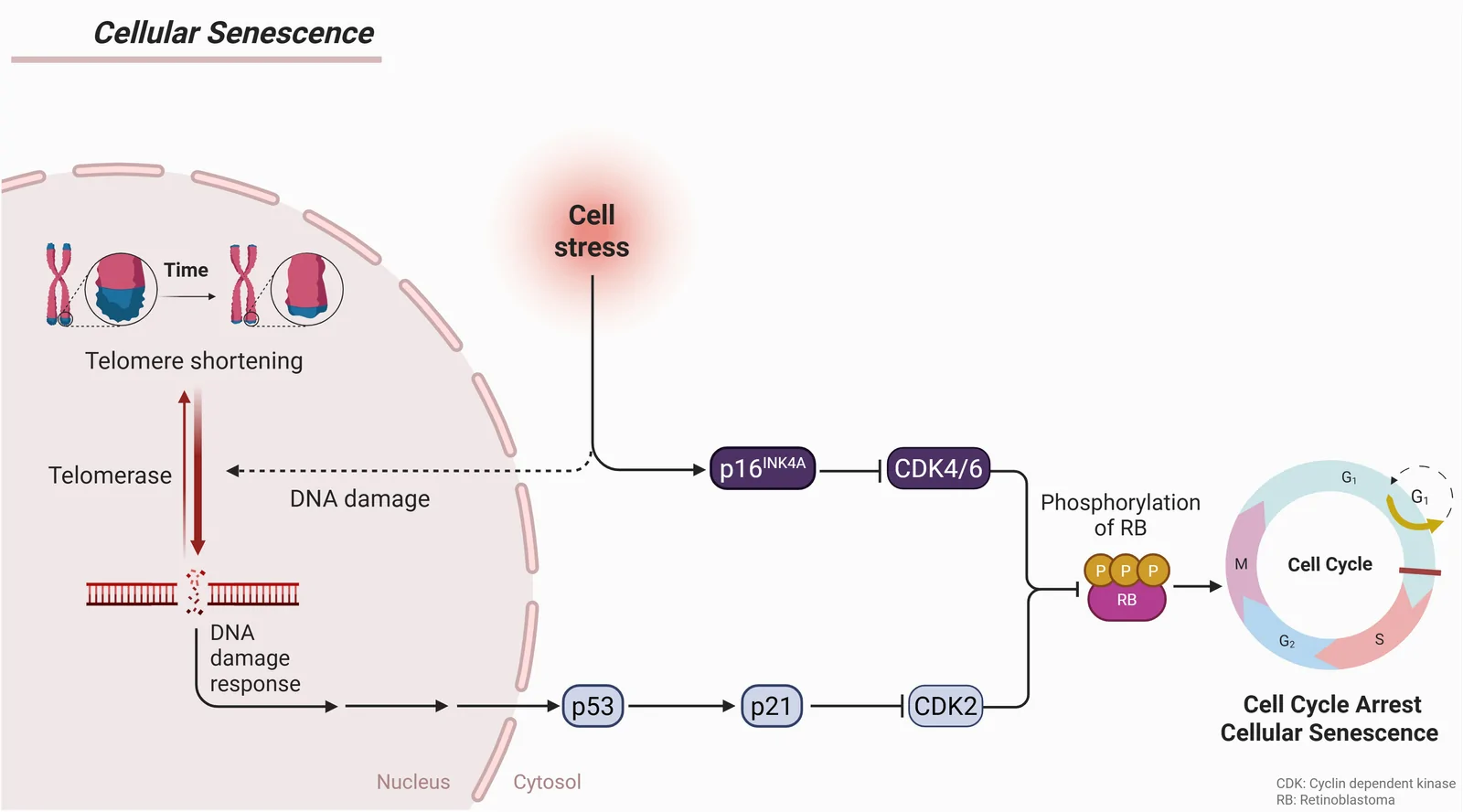
Key cellular senescence pathways. Senescent cells maintain a stable growth-arrested state regulated primarily by the p53/p21 and p16^Ink4a^, retinoblastoma (Rb) pathways, activated in response to cellular stress. These pathways inhibit cyclin-dependent kinases (CDKs), and suppress Rb phosphorylation, enforcing cell cycle arrest state at the G1 phase. CDKs, Cyclin-dependent kinases; Rb, Retinoblastoma protein. Created with Biorender.com.

Moreover, senescent cells are characterized by increased senescent-associated β-galactosidase (SA-β-gal) activity, which is linked to active autophagy and high lysosomal content. SA-β-gal is a lysosomal hydrolase that promotes the breakdown of many β-d-galactoside substrates, including lactose, keratin sulphates, and sphingolipids, within the lysosomal environment (45). Upregulated SA-β-gal activity has been observed in the organs of older adults (78–90 years) and aged animals (24-month-old rats), indicating that cellular senescence is a characteristic of biological aging (46, 47). In isolation or in combination, the presence of SA-β-gal activity, the expression of cell cycle regulators such as p53/p21 and p16^Ink4a^, and telomere shortening have been used to identify senescence cells.

### 2.2 Senescence-associated secretory phenotype

Besides activating cell cycle regulatory proteins, senescent cells can secrete pro-inflammatory mediators to the neighboring cells, which contributes to a series of inflammatory responses. These include immune modulators, inflammatory cytokines, growth factors, and chemokines known as the senescence-associated secretory phenotype (SASP) (48). Notably, these factors released by senescent cells can function in both autocrine (influencing the cell that secretes them) and paracrine effects (as remote endocrine signals, influencing nearby cells) (48). This dual signaling mechanism enhances the spread of senescence through a process called the “bystander effect”. This involves transforming adjacent cells into a senescent state and increasing the overall load of senescent cells within tissues (49). Accumulation of senescent cells over time can negatively impact tissue homeostasis and contribute to aging (**Figure 2**).

**Figure 2:**
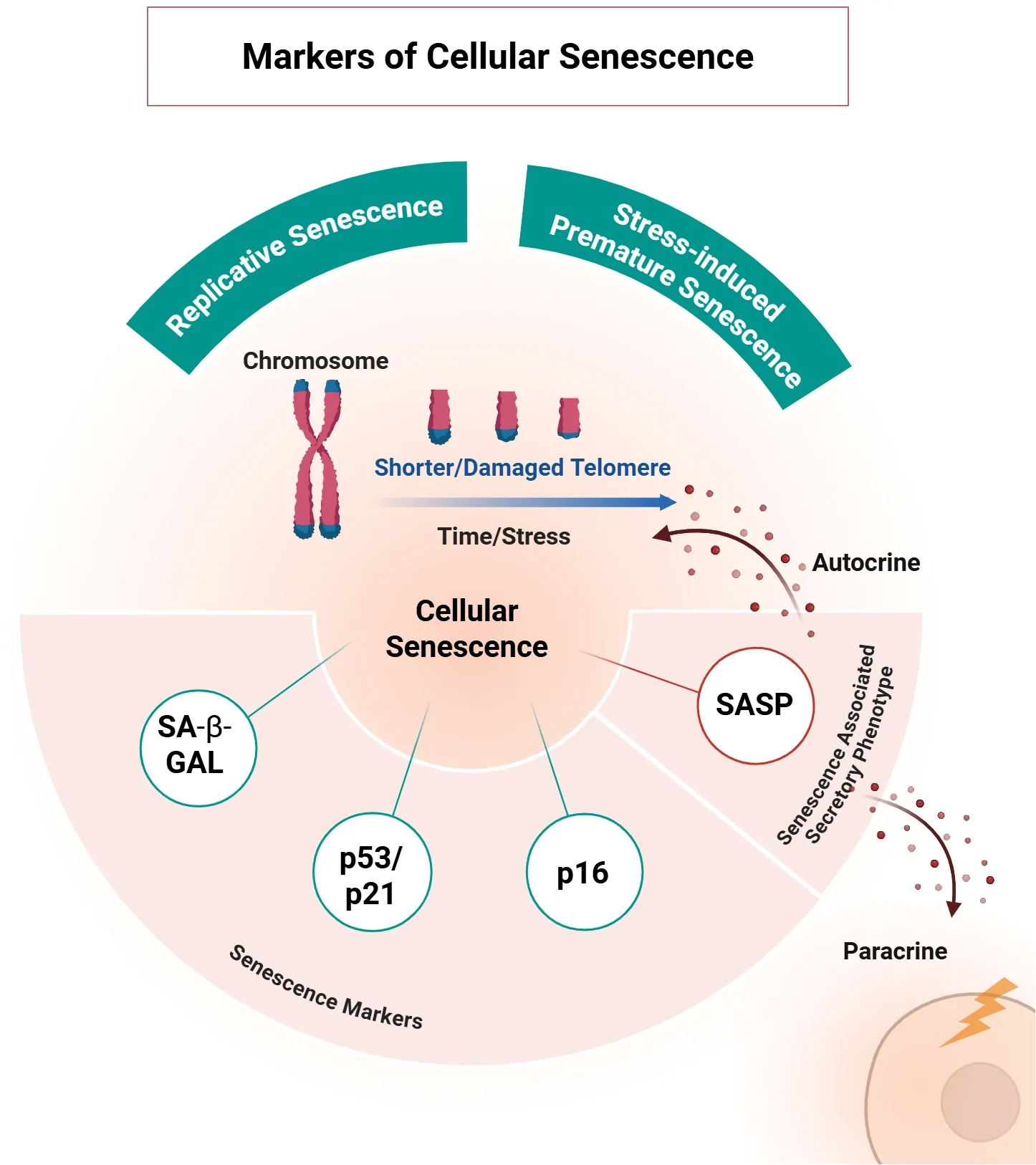
Markers of cellular senescence. Replicative and stress-induced premature senescence result from telomere shortening which triggers permanent cell cycle arrest. Markers of senescent cells include senescence associated β-galactosidase (SA-β-gal), p53, p21, p16^Ink4a^ and senescence-associated secretory phenotype (SASP) secretion. SASP components exert an autocrine or paracrine effect on other cells. Created with Biorender.com.

The SASP includes a plethora of inflammatory cascades. For example, during senescence, increases in both interleukin (IL)-1α and IL-1β proteins activate nuclear factor kappa B (NF-κB) mediated protein transcription, a major feed-forward mechanism in the SASP (50). In brain tissues, the onset of cellular senescence is marked by an increased expression of pro-inflammatory interleukins (IL-6, IL-8, and IL-1α), inhibitory cytokine (IL-10), and matrix metalloproteinases (MMP-1, -2, -3, and -10) (18, 37, 51–53). SASP components that are increased in MS disease models include tumor necrosis factor α (TNF-α), IL-6, IL-8, and IL-1β, vascular endothelial growth factor (VEGF) (54), growth differentiation factor 15 (GDF15) (55), monocyte chemoattractant protein (MCP-1), and chemokine CXC motif ligand 1 (CXCL1) (56).

## 3 Senescence in MS pathology

The pathology of MS is characterized by focal demyelinating plaques in the grey and white matter tissues of the brain and spinal cord (57). In RMS, these lesions are primarily active, marked by the presence of infiltrating peripheral immune cells due to a disrupted blood-brain barrier. Compartmentalized CNS inflammation and slowly expanding lesions (SELs) become more prominent in PMS (57, 58). SELs are characterized by an inactive demyelinated core and severe axonal damage (59, 60), with minimal infiltrating immune cells and are instead surrounded by chronically activated microglia (61). Brain magnetic resonance imaging (MRI) of pwMS shows that the transition between acute inflammatory lesions to SELs becomes more common with advanced aging and longer disease duration (60, 62). SELs generally develop approximately a decade after disease onset and become most prevalent around 50 years of age, corresponding to the typical clinical transition from RMS to PMS (60, 63). Established contributors involved in the development of these lesions include macrophage/microglial activation, chronic oxidative injury, mitochondrial dysfunction, and infections [reviewed in (64)], many of which are linked to eliciting the DNA damage response and triggering cellular senescence (37, 65).

Studies investigating different senescence markers in pwMS are summarized in **Table 1**. Since most CNS neurons are post-mitotic, the applicability of traditional senescence models has been called into doubt. Nevertheless, cumulative stressors and DNA damage driving towards senescence have an active role in demyelinating lesions (37, 66, 67). Human brain autopsies have shown a higher senescence burden, assessed by increased p16^Ink4a^ and p53 expression, in MS white and grey matter tissues compared to healthy controls (37). The higher senescent cell accumulation is associated with disease progression, measured by the time from diagnosis to reaching expanded disability status scale (EDSS) 7.0 (indicative of severe disability) and earlier death (37). Beyond changes in white and grey matter tissues, there is increased accumulation of senescent cells in MS brain lesions compared to other areas (66). While postmortem studies implicate the involvement of cellular senescence in MS brains, the underlying mechanisms remain underexplored. A study reported a senescence phenotype within neural progenitor cells (NPCs) derived from induced pluripotent stem cell (iPSC) lines taken from people with PMS, characterized by increased expression of p16^Ink4a^, p53, and SA-β-gal activity, along with high levels of SASP components such as high mobility group box 1 (67). The soluble factors released from these senescent cells were found to disrupt oligodendrocyte differentiation, leading to inefficient remyelination (67).

**Table 1:** Studies assessing senescence markers in MS.

Tissue/model	Senescence biomarker(s)	Findings in multiple sclerosis	Reference
PBMCs	Telomere length	Shorter telomeres in pwMS (PPMS) compared to healthy controls	(192)
PBMCs	Telomere length	Shorter telomeres in pwMS	(193)
PBMCs	Telomere length and mtDNA copy number	Shorter telomeres in pwMS (RRMS, EDSS 3.5-7.0) compared to healthy controls No changes in mtDNA between pwMS and healthy controls	(194)
PBMCs	Telomere length	Shorter leukocyte telomeres in pwMS are associated with higher EDSS, and brain atrophy	(148)
PBMCs	P16, SASP (Il-6)	Increased p16 in older (> 50 years) pwMS compared to healthy controls No differences in Il-6 expression between pwMS and healthy controls Lower p16 expression in older (> 50 years) pwMS who received anti-CD20 than those not on anti-CD20 therapy P16 and Il-6 are not correlated with EDSS or T25FW	(195)
PBMCs	p53-binding protein 1, γ-H2AX (DNA damage)	γ-H2AX and p53-binding protein 1 were not increased in pwMS (CIS, RRMS) compared with healthy controls No correlation of γ-H2AX and p53-binding protein 1 with MRI lesion burden or EDSS	(196)
Peripheral blood CD4+ and CD8+ T lymphocytes	Telomere length and thymic T cell production	No changes in telomere length in pwMS (RRMS) compared to healthy controls Thymic T cell production was decreased in pwMS (RRMS) compared to healthy controls	(197)
Blood DNA	Telomere length	Shorter telomeres in pwMS (RRMS, SPMS, PPMS) compared to healthy controls Shorter telomeres are associated with high EDSS in pwMS (RRMS, SPMS, PPMS)	(198)
Blood DNA	Telomere length	Shorter telomeres in pwMS (RRMS and PPMS) compared to healthy controls Shorter telomeres predict higher rate of conversion from RRMS to SPMS	(199)
Blood DNA	Telomere length	At baseline, shorter telomeres were found in pwMS (PMS) compared to pwMS (RRMS) pwMS treated with high-efficacy DMT had shorter telomeres Telomere lengths were positively correlated with disease duration and negatively with EDSS, 9HPT, smoking, brain lesion volumes, and memory performance in pwMS	(200)
Postmortem brain tissues	P16	Increased p16 in the lesion core compared to other areas in the MS brain Increased p16 in the cortex and WM in MS brain compared to control tissues	(66)
Postmortem brain tissues and CSF	P16, p53-binding protein 1, SASP (Il-6, Il-10, TNF-α, MIF, CCL25, CXCL2, CCL7, CCL3)	P16, p53-binding protein 1 expression increased in MS brain tissues (NAWM, WM, GM) compared to healthy controls Increased SASP in CSF in pwMS compared to healthy controls Increased p16 is associated with shorter time to EDSS 7.0 and age at death in PMS brain tissues	(37)
Postmortem brain tissue and iPSC-derived neural models (glia-enriched organoid)	P16, SA-β-gal	Increased p16 in MS brains compared to healthy controls Increased SA-β-gal in the organoid model after MS CSF exposure Tolebrutinib (BTK inhibitor) treatment reduced the SA-β-gal increase	(72)
Cerebral organoid from iPSC-derived from pwMS (RRMS, PPMS, SPMS)	P21, p53	Reduced p21 in pwMS (PPMS, SPMS) compared to healthy controls No differences in p53 expression between MS and healthy controls	(69)
Postmortem brain tissue and neural progenitor cells (NPCs) derived from pwMS (PPMS)	P16, SA-β-gal p53	No changes in p16 expression in MS brains (white matter and lesions) P16, SA-β-gal, and p53 are increased in NPCs derived from pwMS compared to non-disease controls	(67)
Human periodontal ligament stem cells	SA-β-gal, p21, p16	No senescence markers elevated at baseline culture phase/passage Following prolonged culture phase/passaging, increased SA-β-gal, p21, p16 expression in cells taken from pwMS compared with early-passage cells	(201)
Bone marrow-derived mesenchymal stem cells (MSCs)	P16, Telomere length	Increased p16 positive cells in bone marrow biopsies taken from pwMS No changes in telomere length between pwMS (PMS) and healthy controls. However, telomere length shortening is faster after cell passaging in MSCs taken from pwMS	(202)
Skin fibroblasts	P16, p21, p53	P53 is increased in pwMS compared to healthy controls P21 expression is increased in irradiated MS fibroblasts compared to controls with non neurological disorders No differences in p16 expression	(70)
Induced neural stem cells (iNSCs) fibroblasts derived from pwMS (PMS)	SA-β-gal, p21, p16, SASP (GDF15)	Increased SA-β-gal, p21, p16, GDF15 in iNSCs from PMS fibroblast compared to healthy controls	(71)
Human- International Multiple Sclerosis Genetics Consortium	Telomere length	Positive association between leukocyte telomere length and risk of MS	(203)

BTK, Bruton’s tyrosine kinase; CIS, clinically isolated syndrome; CSF, cerebrospinal fluid; EDSS, expanded disability status scale; GDF15, growth differentiation factor 15; GM, grey matter; IL, interleukin; iPSC, induced pluripotent stem cells; MIF, macrophage migration inhibitory factor; mtDNA, mitochondrial DNA; MRI, magnetic resonance imaging; MS, multiple sclerosis; MSC, mesenchymal stem cell; NAWM, normal-appearing white matter; NPC, neural progenitor cells; PBMC, peripheral blood mononuclear cells; PMS, progressive multiple sclerosis; PPMS, PMS, progressive multiple sclerosis; pwMS, persons with multiple sclerosis; RRMS, relapsing-remitting multiple sclerosis; SASP, senescence-associated secretory phenotype; SA-β-gal, senescence-associated beta-galactosidase; SPMS, secondary progressive multiple sclerosis; TNFα, tumor necrosis factor α; T25FW, Timed 25-Foot Walk; WM, white matter; 9HPT, 9 Hole Peg Test.

Another common marker is p21, a member of the CDKI and essential component for p53-mediated cell cycle arrest, which was found to be one of the differentially expressed genes in the cerebrospinal fluid (CSF) of pwMS (68). However, there does not appear to be a consistent p21 pattern across tissue levels in pwMS (69–71). For example, in a cerebral organoid model from iPSC derived from pwMS, p21 levels were reduced compared to controls (69). In contrast, irradiated skin fibroblasts and induced neural stem cell (iNSC) fibroblasts taken from pwMS showed increased levels of p21 compared to controls (70, 71). Studies assessing SA-β-gal in MS human models are more consistent and report an increase in SA-β-gal activity in NPCs derived from iPCs taken from pwMS and cerebral organoids exposed to MS CSF (67, 72). However, no specific single marker can reflect the senescence burden. The limited findings of senescence marker trends in pwMS highlight the need for a multi-marker approach to accurately track senescence in MS. Nonetheless, these findings indicate that cellular senescence contributes to MS pathogenesis and may have a deleterious effect on the remyelination process in PMS.

The elevated SASP in MS characterized by proinflammatory cytokines, chemokines, growth factors, and reactive oxygen species may self-reinforce senescence or affect neighboring cells. Increased accumulation of SASP markers has been observed in the CSF, serum, and/or plasma of pwMS. A study by Itorralba et al. (73) analyzed the levels of CSF cytokines (common SASP components) to predict the risk of severe disability in pwMS measured by EDSS and Multiple Sclerosis Severity Score (MSSS). Higher CSF levels of IL-1β, IL-10, TNF-α, and IFN-γ were seen in pwMS, while IL-6 was associated with increased EDSS and MSSS (73). In people with RMS, TNF-α levels were associated with brain and spinal cord lesions, while IL-1β levels inversely correlated with vitamin D levels (74). In PMS, TNF-α and IL-8 proinflammatory mediators were linked to higher EDSS scores, and IL-15 and TNF-α were increased in the presence of gadolinium-enhancing lesions on MRI (75). Notably, these inflammatory markers have been previously linked with the neurodegenerative cascade involved in Alzheimer’s disease (AD) and Parkinson’s disease (76–80). In addition to serving as a clinical biomarker for disease severity, release of these SASP components can be connected to the accumulation of senescent cells in neurodegenerative diseases, which suggests that cellular senescence actively drives chronic inflammation and neurodegeneration, a possibility that warrants further exploration in MS.

Another SASP component is GDF15, which is part of the transforming growth factor beta superfamily (81). Recent reports indicate GDF15 serves as a novel biomarker for aging, cellular senescence, and mitochondrial disease (82–85). Several studies demonstrate GDF15 to be a biomarker of PMS (86, 87), where elevated levels are presumably connected to the neurodegenerative processes within the CNS, which in turn may promote demyelination and axonal degeneration (83, 87). Moreover, GDF15 levels are elevated in people with RMS that are clinically and radiologically stable and are correlated with EDSS scores (87). This supports the notion that subclinical neurodegeneration begins earlier in clinically stable pwMS and predicts faster disability accumulation where degenerative processes dominate over inflammatory flares (55).

## 4 Immunosenescence in MS

Under normal physiological conditions, the immune system recognizes and eliminates senescent cells. However, natural aging is associated with functional decline in the immune system (immunosenescence), which impairs the clearance of senescent cells (88). Accumulation of senescent cells sustains SASP secretion and contributes to “inflammaging”, which is characterized by increased secretion of inflammatory cytokines (partly in response to infections) and increased levels of chronic, low-grade inflammation, resulting in a pro-inflammatory phenotype in older people that appears to occur prematurely in pwMS (89, 90). Such deleterious changes are further enhanced by the “bystander effect” caused by the actions of SASP released from senescent cells and contribute to the onset of senescence in healthy cells, establishing a vicious cycle of inflammation and immunosenescence (89). Age-related immune changes are driven by thymic involution (age-related atrophy of the thymus), inflammaging, T and B cell senescence (**Figure 3**) (89, 90).

**Figure 3:**
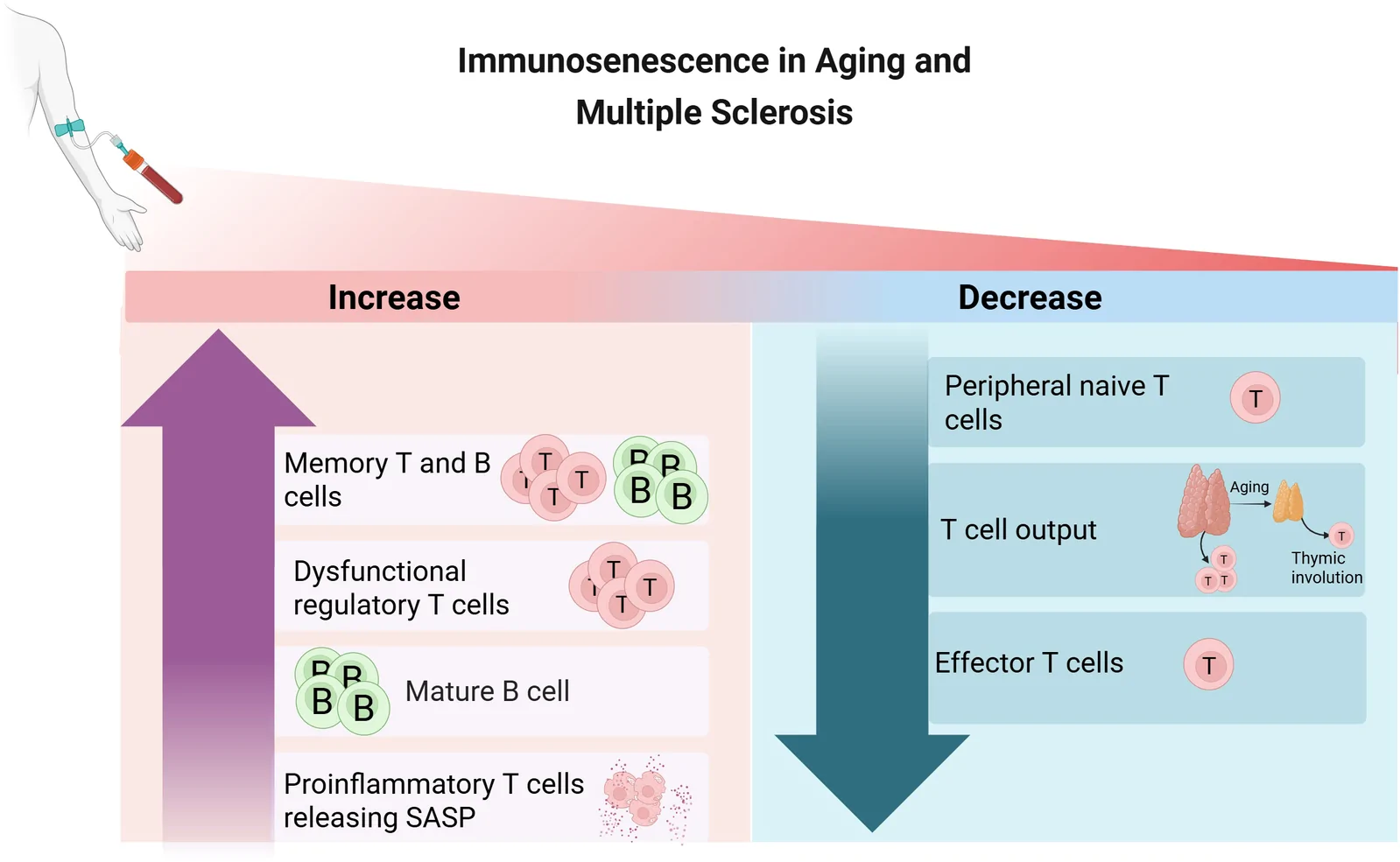
Key changes in peripheral immunosenescence with aging and MS disease. SASP, senescence associated secretory phenotype. Created with Biorender.com.

It is important to distinguish immunosenescence from immune exhaustion. Immune exhaustion is an antigen-driven state resulting from persistent viral stimulation and characterized by progressive loss of effector function, whereas senescent cells remain functionally active and secrete SASP factors (91, 92).

### 4.1 Role of T cells and B cells in immunosenescence

Immunosenescence affects both adaptive and innate immunity. Among immune cell populations, T cell alterations are the most widely documented in immunosenescence, characterized by reduced peripheral naïve T cells, dysfunctional regulatory T cells, and accumulation of terminally differentiated memory T cells (93). Terminally differentiated T cells include both CD4+ and CD8+ T cells, which acquire senescence traits with aging and chronic antigen exposure (94). They exhibit hallmark features of cellular senescence, including telomere shortening, reduced proliferative capacity, and secretion of pro-inflammatory SASP factors that may contribute to tissue damage (95, 96). The main immune phenotype alteration reported for pwMS is overactivity of pro-inflammatory T cells (both CD4+ and CD8+) as key drivers of relapses (97). PBMCs from pwMS show higher levels of senescent CD4^+^CD28^−^ T cells and premature immune system senescence (98). Moreover, circulating cells taken from pwMS showed excessive pro-inflammatory traits, including an increase in the cytokines TNF-α, IL-12, IL-6, IL-23, and IL-1β that drive differentiation and senescence of T cells (99). One potential cause of unregulated pro-inflammatory T cells is weak regulatory T cells (Treg) and the resistance of these effector cells to regulatory controls. Evidence reports defects in Treg cells in pwMS, characterized by low forkhead box P3 (FOXP3), a master Treg transcription factor, and poor inhibition of pro-inflammatory T cells (100). Aged T cells often produce senescence markers and lose expression of CD4^+^CD28^−^ effector memory T cells, which represents a hallmark shared between physiological aging and MS (101). Moreover, naïve T cell pools progressively decline in pwMS as a result of thymic involution and repeated stimulation of preexisting myelin-specific T-cell clones (102). This leads to reductions in naïve CD4+ and CD8+ T cells, with diminished CD8+ naïve T cells in PMS, consistent with a senescent immune profile (103). While not commonly used in clinical practice, CD4/CD8 ratios are considered an indicator of active RMS disease, primarily driven by a decrease in the number and percentage of peripheral CD8+ T cells (104).

The B cell compartment in MS exhibits immunosenescent alterations similar to those in T cells. Multiple studies demonstrate that pwMS display increased mature and senescent B cell phenotypes typical of accelerated aging (101). These include double-negative IgD^−^ CD27^−^ and CD10^−^ CD21^−^ B cells, accompanied by a relative loss of naïve or early memory B cells (105). This is prominent in pwMS treated with anti-CD20 therapies, where significant depletion of naïve and non-switched memory B cells occurs, with expansion of senescent B cell phenotypes such as double-negative activated memory (CD21^low^ CD27^+^), tissue-like memory (TLM, CD21^low^ CD27^−^), and apoptosis-prone (CD95^+^) B cells (105, 106). These mature and senescent B cells are also programmed to overproduce pro-inflammatory cytokines, including lymphotoxin and TNF-α, further mediating the bystander effect of disease-relevant proinflammatory T cells (107).

### 4.2 Epstein-Barr virus, cytomegalovirus and herpes simplex virus-1 in immunosenescence

A growing body of evidence indicates that multiple genetic and environmental factors affect immunosenescence. Epstein-Barr virus (EBV), cytomegalovirus (CMV), and herpes simplex virus type 1 (HSV-1) are members of the herpesvirus family that cause lifelong latency in the human host which is typically asymptomatic (108). EBV is strongly associated with MS, and an immunopathological response to EBV may be a major determinant of CNS damage in pwMS (109–111). Activated EBV-infected B cells express a limited set of “latency II” genes called Epstein-Barr nuclear antigen 1 (*Ebna1*), latent membrane protein 1 (*Lmp1*), and latent membrane protein 2A (*Lmp2A*) (112). In this state, *Lmp1* mimics CD40 signaling, driving inflammatory NF-κB signaling, and enhancing B-cell survival (113). These B cells infiltrate CNS perivascular spaces, recruit T cells, and drive chronic inflammation. Chronic antigen stimulation leads to dysfunction of EBV-specific CD8^+^ T cells, which allows latent EBV to be reactivated or persist in B cells, causing ongoing neuroinflammation (114). Supporting evidence indicates dominance of CD8^+^ T cells in CNS infiltrates, EBV-specific CD8^+^ T-cell enrichment in the CSF of pwMS (115, 116), and elevated peripheral EBV lytic antigen-specific CD8^+^ T cells during active disease (117). Chronic antigen stimulation of EBV drives telomere shortening primarily in EBV specific CD8+ T cells (114, 118). It is worth mentioning that these cells continue to be exposed to EBV antigen without upregulation of the telomerase enzyme, suggesting that chronic EBV exposure leads to replicative senescence in CD8+ T cells (119). Taken together, EBV-specific CD8^+^ T cells exhibit significant telomere shortening, leading to a senescent-like state, mirroring T-cell dysfunction in older people (120).

Recurrent antigenic stimulation from CMV accelerates biological aging (121). Reduced leukocyte telomere length has been shown in people with CMV (122). CMV latently induces massive clonal expansion of terminally differentiated T cells, characterized by shorter telomeres and senescent cells (122, 123). Moreover, CMV promotes inflammaging by stimulating SASP production through inflammatory pathways like NF-κB activation and the release of IL-6, IL-8 and TNF-α (121, 123). The accumulation of senescent-like CD8+ T cells, which are less effective and have pro-inflammatory mediator secretion, correlates with MS progression, particularly with aging (124, 125).

Among environmental triggers, herpes simplex virus type 1 (HSV-1) has also been implicated in MS (126). HSV-1 forms a lifelong latency in sensory neurons of the trigeminal and dorsal root ganglia. Periodic reactivation of the virus sustains a population of HSV-1-specific memory T cells that restrain viral gene expression, maintaining repeated antigenic stimulation within a narrow T-cell repertoire (127). HSV-1 has been detected in brain tissues, CSF, and the blood of pwMS in early virological surveys (128, 129), and viral reactivation has been reported during acute relapse episodes in RMS (130). This is supported by postmortem brain tissue analysis in which HSV-1 DNA was more frequently detected in active than in inactive demyelinating plaques (131), placing the virus at sites of ongoing inflammatory activity. Experimentally, asymptomatic HSV-1 brain infection combined with EAE was found to induce cellular senescence in the murine CNS, evidenced by increased p21, MMP-12, CCL2 and CXCL2, providing a mechanistic link between viral persistence and the neuroinflammatory environment (132).

As mentioned previously, stress-induced premature senescence may occur independently of biological age. Chronic antigen stimulation increases inflammatory cytokines and oxidative stress, and promotes premature immunosenescence in pwMS. Reported features of immunosenescence in MS include reduced thymic output, contraction of naïve T-cell pool, along with expansion of highly differentiated CD4+CD28− T cells and increased senescent phenotypes by CD8+ T cells. In parallel, persistent neuroinflammation may induce senescence-like changes in CNS-resident cells, including neurons (133), microglia, astrocytes, oligodendrocytes, and oligodendrocyte progenitor cells (134). These changes could contribute to demyelination and neurodegeneration even in relatively young pwMS. However, the lack of specific biomarkers makes it difficult to distinguish premature senescence from accelerated biological aging in pwMS. Valid biomarkers differentiating stress-induced senescence from normal aging would clarify the role of premature senescence in MS progression (135).

## 5 Brain atrophy and senescence in MS

Brain atrophy involves a gradual loss of brain volume, caused by neuronal loss and degradation of synapses (136). Brain atrophy is often used as a biomarker for tracking the progression of many neurological diseases, including MS. While gradual loss of brain volume happens over the normal aging process, accelerated brain atrophy is a common hallmark of MS. MS-specific atrophy occurs across all stages of MS and is observed even in the earliest stages of the disease (137, 138). Azevedo et al. studied the percentage of brain volume change over time and found that by the age of 30, the majority of atrophy observed was due to MS disease rather than normal aging, indicating that brain atrophy at a younger age is driven primarily by MS disease-related processes rather than the normal aging process (139). Brain atrophy occurs at a higher rate per year in pwMS than healthy controls (140) and becomes more severe as the disease progresses (141). Moreover, the rate of brain atrophy is linked with disability progression (increase in EDSS ≥ 1.0) and a higher likelihood of transitioning to SPMS (141, 142). For example, cerebellar cortical volume was a sufficient marker to predict the likelihood of people with RMS developing PMS (141).

Theories behind brain atrophy in pwMS could be related to increased senescence burden and neuroinflammation. One of the key senescence markers, p16^INK4a^, was found to promote age-dependent changes, and that clearance of p16^INK4a^ positive senescent cells preserves cognitive function (143, 144). In a human brain post-mortem study, astrocytes from older age controls expressed more p16^Ink4a^ than fetal autopsies (18). Senescent human astrocytes are characterized by morphology changes and increased levels of senescence markers such as p53, p21, p16^Ink4a^ along with activation of SASP pathways such as NF-κB and IL-6 (145, 146). The release of the SASP mediators such as NF-κB is linked with white matter degeneration (147). The enriched senescence and inflammatory environment in the brain may contribute to increased EDDS levels in pwMS relative to their chronological age. This notion is supported by a study finding shorter leukocyte telomere length was associated with higher EDSS scores and brain atrophy in pwMS (148).

## 6 The relationship between disease modifying therapies and aging in MS

Therapeutic targeting of peripheral immune cell activity with immunodepleting or lymphocyte anti-trafficking drugs is effective in suppressing inflammation in MS; however, their efficacy against ongoing disease progression is relatively limited (149). Disease-modifying therapies (DMTs) are effective in active MS with relapses and/or new MRI activity (150, 151), but their benefit in older pwMS is less pronounced. For example, in the ORATORIO-HAND clinical trial, the therapeutic advantage of ocrelizumab, one of the approved DMTs in PPMS, was most significant in pwMS age 55 or younger, while those over 55 had less benefit (152). Furthermore, a meta-analysis of 38 MS clinical trials showed that the benefit of DMT on MS disability progression strongly decreased with advancing age (>53 years) (153). There is also evidence from other clinical trials supporting the notion of reduced DMT efficacy in older pwMS (154, 155). These observations raised a discussion of whether to continue or discontinue DMTs in older pwMS (age 55 and older) and led to the DISCOMS trial assessing the risk of disease activity following DMT discontinuation, finding that 12.2% in the discontinue group developed a clinical relapse and/or new MRI activity compared to 4.7% of the continue group, precluding the conclusion that discontinuation is non-inferior to continuation (156). Subsequently, the DOT-MS trial reported that DMT discontinuation increased the risk of recurrent inflammatory disease activity with 17.8% that discontinued therapy developing new inflammatory activity (MRI and/or clinical relapse), leading to the trial being terminated prematurely (157). It remains uncertain when the appropriate time is to discontinue DMT in older pwMS, for which the answer may pertain more to biological age than chronological age in a given individual.

Immunosenescence and the adaptive immune system could play a role in reducing the efficacy of DMTs in older pwMS. Biological aging leads to thymic involution, resulting in reduced peripheral naïve T cells and expansion of existing memory T cells (93). In active MS, T cells play a vital role in initiating and maintaining neuroinflammation, and with repeated activation, T cells become old characterized by reduced expression of CD28 (98). Similarly, senescence affects the production of naïve or early memory B cells, leading to a depletion of naïve B cells and the expansion of diverse B cell receptor repertoires. These senescent B cells are characterized by higher levels of signaling via CD80, CD86, and CD40, which lead to increased inflammatory T cell responses and worsening MS disease (158, 159). In this senescence, the quality of the humoral immune system response diminishes rather than the quantity, resulting in the production of less effective antibodies (160). Long-term treatment with DMTs targets peripheral naïve T cells and B cells, leading to reduced diversity of the immune system, which could explain why the combination of aging and DMTs exacerbates adaptive immunosenescence in MS. Conceptually, if disease progression in PMS was primarily mediated by adaptive immune response, the effectiveness of DMTs should have remained stable or improved in older pwMS. Instead, DMTs do not appear particularly effective for non-active PMS, suggesting that factors beyond peripheral immune responses should be considered, particularly regarding compartmentalized inflammation, which may drive disability (57). Other therapeutics such as the new Bruton’s tyrosine kinase (BTK) medications, which target components of the innate immune system including microglia (161–163), or senotherapeutic agents could be effective for managing PMS.

## 7 Senotherapeutics

The beneficial effects observed with senescence medications have validated the association between cellular senescence accumulation and age-related diseases in animal models (164–166). Interventions reducing the burden of senescent cells may alleviate age-related diseases and extend the lifespan (167). Preclinical and clinical trials have shown positive results for senescence-targeting medications in longevity extension and age-related diseases, including AD, diabetic kidney disease, and pulmonary fibrosis (168–170). Senescence medications include two types: 1) senolytics, which selectively target senescent cells and actively remove them; and 2) senomorphics, which inhibit the release and consequences of senescent cells and block SASP release, thus maintaining the environment (171).

### 7.1 Senolytics

Dasatinib and Quercetin (D+Q) are known as a first-in-class senolytic medication combination triggering the death of senescent cells (167). Dasatinib (D) is a tyrosine kinase inhibitor, while Quercetin (Q) targets B-cell lymphoma 2 (BCL-2)/p53 to inhibit the anti-apoptotic pathways and eliminate senescent cells (172, 173). Combination D+Q selectively targets senescent cells that have anti-apoptotic pathways that enable the cell to survive despite the release of SASP components (172, 173). Early trials of D+Q have shown improved physical function in idiopathic pulmonary fibrosis and successful penetration of dasatinib into the brains of people with AD (170, 174). The safety and tolerability of D+Q have been confirmed in several preclinical and clinical trials (165, 167, 170, 174, 175). However, one recent preclinical study reported that D+Q administration caused demyelination in the corpus callosum of young and aged naïve mice (176). Thus, the long-term safety of repeated senolytic treatment remains uncertain. Longer-term trials are needed to further ascertain the safety of D+Q, especially in neurodegenerative diseases such as AD and MS.

Another senolytic medication candidate is Navitoclax (ABT263), which inhibits the anti-apoptotic BCL-2 protein, inducing apoptosis in senescent cells (177). While Navitoclax exhibits poor CNS penetration clinically, high dose administration in mice has been shown to clear senescent glial cells (168). Administering Navitoclax prevents the upregulation of senescence associated genes and cognitive decline in a tau-mediated mouse model (168, 177). Initial clinical trials are currently being conducted to assess the safety and effectiveness of senolytic medications in treating various chronic conditions linked to aging (178). STAMINA and SToMP-AD are two recent clinical trials exploring the potential use of D+Q in mitigating the progression to neurodegenerative diseases and preventing AD, respectively (174, 179). Notably, there is an ongoing clinical trial (NCT07270120) from Ohio State University testing combination D+Q in SPMS, hypothesizing that D+Q will improve physical and cognitive function and reduce neurodegenerative and senescence biomarkers (https://clinicaltrials.gov/study/NCT07270120).

### 7.2 Senomorphics (SASP modulation)

Another strategy to target senescent cells is through SASP modulation. SASP modulators or senomorphic drugs reduce the release of SASP component signaling and attenuate their deleterious consequences without killing the senescent cells (180). This approach might disrupt the proinflammatory nature of senescent cells and attenuate pathways related to aging and age-associated diseases (180). Modulating SASP can be accomplished by targeting and blocking pathways involved in inflammation and cell proliferation, such as NF-κB, and the mammalian target of rapamycin (mTOR) pathways. Various compounds that affect NF-κB signaling and have been found to lower SASP production, such as metformin, which is used for type 2 diabetes (181). Metformin likely suppresses SASP through the I kappa B kinase (IKK)/NF-κB inhibition and delays cellular senescence through the adenosine monophosphate-activated protein kinase (AMPK)/mTOR modulation (181). A study in monkeys found that metformin had a neuroprotective effect with a six-year reduction in brain aging and improved cognitive performance (182). In addition, a retrospective study found that people aged 45 and younger with type 2 diabetes exposed to antihyperglycemic agents (including metformin) had a reduced risk of developing MS compared to those over age 45, revealing a possible prophylactic effect of antihyperglycemic agents against MS onset (183). There is an ongoing trial, MACSiMiSE-BRAIN, investigating the potential use of metformin as an add-on therapy in people with PMS, targeting neurodegeneration and remyelination pathways where senomorphic suppression could slow down the disability progression (184).

Moreover, the mTOR pathway promotes SASP production by increased translation of IL-1α, the upstream regulator of NF-κB, which indirectly stabilizes many cytokine mediators (185). In the context of MS, treatment with rapamycin had clinical therapeutic significance (67, 186). In people with RMS, six months of rapamycin treatment was linked to clinical and radiological improvement, where EDSS scores decreased in 50% of participants and MRI showed reductions in lesion volume (186). In NPCs from induced iPSC lines derived from pwMS, rapamycin attenuated the release of the senescence markers p16^Ink4a^ and SA-β-gal, ameliorated cellular aging, and enhanced oligodendrocyte progenitor cell differentiation (67). Additional evidence has been gleaned from animal models where rapamycin reduced inflammatory SASP production, IFN-γ, and IL-17 in the experimental autoimmune encephalomyelitis mouse model of MS (187). Beyond MS, several studies report that rapamycin treatment increases lifespan and delays certain aging-related dysfunctions in mice (188, 189).

Because senescent cells develop slowly, preclinical data shows that even 30% clearance of senescent cells delivers major benefits that reduce cellular senescence and delay age-related pathologies such as frailty, atherosclerosis, osteoarthritis, glaucoma, chronic obstructive pulmonary disease, and type 2 diabetes (190). Senolytic therapies may offer advantages over senomorphic therapies for several reasons. First, senolytics eliminate the root cause (senescent cells), preventing them from releasing SASP factors. Second, senolytics use intermittent dosing without the need for continuous administration, which is the case for senomorphics. This is because senolytics follow the “hit and run” strategy, which allows time for the targeted senescence cells to accumulate and develop their pathological SASP effects. This intermittent strategy also reduces off-target risks and drug resistance events in older people. While the p16-INK-ATTAC mice that are engineered for targeted removal of p16^Ink4a^ positive senescent cells, had no obvious harmful consequences following senolysis activation, the long-term safety of repeated senolytic treatment remains to be determined. The current FDA-approved compounds such as dasatinib, quercetin, metformin, and navitoclax offer potential senotherapeutic properties based on promising preclinical and clinical data. Repurposing these medications for MS may serve as a potential strategy to meet the urgent need of delaying or preventing MS disease progression.

## 8 Future perspectives

Traditionally, MS begins with frequent acute inflammatory relapses that decrease with age. The progression from inflammatory to neurodegenerative phases underscores the complex and dynamic pathophysiology of MS. The link between aging and disability progression points toward age-related mechanisms as key drivers in PMS. Biological aging in MS drives the pathophysiological transition from peripheral immune cell biology to chronic lesions with compartmentalized inflammation in PMS. Cellular senescence within both the immune system and CNS influences changes in the pathophysiology of MS disease. However, these mechanisms are not exclusively determined by chronological age. Sustained inflammation, and viral exposure (e.g., EBV, CMV and HSV-1) may induce premature senescence in peripheral and CNS-resident cells in those whose chronological age would not predict it.

The involvement of senescence in the pathology of MS has recently been established; however, it is still in its infancy and the exact effect of cellular senescence in different cellular contexts is not fully explored. For example, senescent immune cells promote chronic inflammation through SASP mediated signaling by the “bystander effect”, whereas senescence in oligodendrocyte progenitor cells negatively affects the remyelination process in PMS (133, 134). Thus, the contribution of cellular senescence across different CNS cell populations, including neurons (133), microglia, astrocytes, oligodendrocytes, oligodendrocyte progenitor cells (134) and peripheral immune cells, remains largely unknown and warrants further investigation. Single cell transcriptomics studies are needed to define which cell populations become senescent, and which are worth targeting in PMS.

The exploration of cellular senescence pathways in MS could highlight the clinical significance of biological aging clinically. For instance, pwMS could be stratified based on their biological age to predict disease outcomes and treatment responses. No single marker captures senescent burden. Biomarkers such as telomere shortening, SA-β-gal, p16, p53/p21, and SASP components may quantify senescence burden in pwMS. Correlating these markers with clinical and MRI measures is paramount and should be a priority in the MS field (191). Furthermore, current DMTs are effective in reducing acute relapses, but less effective in older pwMS primarily because they target the adaptive immune system, which may not be the sole driver of PMS. Given the development of clinically applicable senotherapeutics, work in this area presents a promising path to delay, or perhaps even slow, MS progression.

In conclusion, accumulation of senescent cells may contribute to disease progression in pwMS, although its effects may vary among individuals. Validating biomarkers that capture senescent burden and identifying which cell populations drive this process represent critical next steps towards understanding progression in pwMS.

## Glossary

9HPT: Nine-Hole Peg Test
AD: Alzheimer’s disease
AMPK: Adenosine monophosphate-activated protein kinase
ATM: Ataxia-telangiectasia mutated
ATR: Ataxia-telangiectasia and Rad3-related
BCL-2: B-cell lymphoma 2
BTK: Bruton’s tyrosine kinase
CDKI: Cyclin-dependent kinase inhibitors
CDKs: Cyclin-dependent kinases
CIS: Clinically isolated syndrome
CMV: Cytomegalovirus
CNS: Central nervous system
CSF: Cerebrospinal fluid
CXCL: Chemokine (C-X-C motif) ligand
DISCOMS: Discontinuation of Disease-Modifying Therapies in Multiple Sclerosis
DMTs: Disease-modifying therapies
EBNA1: Epstein-Barr nuclear antigen 1
EBV: Epstein-Barr virus
EDSS: Expanded Disability Status Scale
FDA: Food and Drug Administration
FOXP3: Forkhead box P3
GDF15: Growth differentiation factor 15
GM: Grey matter
HSV-1: Herpes simplex virus type 1
IFN: Interferon
Ig: Immunoglobulin
IKK: I kappa B kinase
IL: Interleukin
iNSC: Induced neural stem cell
iPSC: Induced pluripotent stem cell
LMP: Latent membrane protein
LOMS: Late-onset multiple sclerosis
MACSiMiSE: Metformin Add-on Clinical Study in Multiple Sclerosis
MCP-1: Monocyte chemoattractant protein-1
MIF: Macrophage migration inhibitory factor
MMP: Matrix metalloproteinase
MRI: Magnetic resonance imaging
MS: Multiple sclerosis
MSC: Mesenchymal stem cell
MSSS: Multiple Sclerosis Severity Score
mtDNA: Mitochondrial DNA
mTOR: Mammalian target of rapamycin
NAWM: Normal-appearing white matter
NF-κB: Nuclear factor kappa-light-chain-enhancer of activated B cells
NPCs: Neural progenitor cells
PBMCs: Peripheral blood mononuclear cells
PMS: Progressive multiple sclerosis
POMS: Pediatric-onset multiple sclerosis
PPMS: Primary progressive multiple sclerosis
pwMS: Persons with multiple sclerosis
RMS: Relapsing multiple sclerosis
RRMS: Relapsing-remitting multiple sclerosis
SA-β-gal: Senescence-associated beta-galactosidase
SASP: Senescence-associated secretory phenotype
SELs: Slowly expanding lesions
SPMS: Secondary progressive multiple sclerosis
T25FW: Timed 25-Foot Walk
TNF-α: Tumor necrosis factor alpha
VEGF: Vascular endothelial growth factor
WM: White matter

## References

[1] King R 3rd Multiple Sclerosis International Federation . Atlas of MS 3rd Edition: Mapping Multiple Sclerosis Around the World. London: Multiple Sclerosis International Federation. (2020).

[2] Oh J Vidal-Jordana A Montalban X . Multiple sclerosis: clinical aspects. Curr Opin Neurol. (2018) 31:752–9. doi: 10.1097/WCO.0000000000000622 30300239

[3] Cree BA Gourraud PA Oksenberg JR Bevan C Crabtree-Hartman E University of California SFMSET . Long-term evolution of multiple sclerosis disability in the treatment era. Ann Neurol. (2016) 80:499–510. doi: 10.1002/ana.24747 27464262 PMC5105678

[4] Confavreux C Vukusic S . Age at disability milestones in multiple sclerosis. Brain. (2006) 129:595–605. doi: 10.1093/brain/awh714 16415309

[5] Trojano M Liguori M Bosco Zimatore G Bugarini R Avolio C Paolicelli D . Age-related disability in multiple sclerosis. Ann Neurol. (2002) 51:475–80. doi: 10.1002/ana.10147 11921053

[6] Tutuncu M Tang J Zeid NA Kale N Crusan DJ Atkinson EJ . Onset of progressive phase is an age-dependent clinical milestone in multiple sclerosis. Mult Scler. (2013) 19:188–98. doi: 10.1177/1352458512451510 22736750 PMC4029334

[7] Niccoli T Partridge L . Ageing as a risk factor for disease. Curr Biol. (2012) 22:R741–52. doi: 10.1016/j.cub.2012.07.024 22975005

[8] Zhang Y Atkinson J Burd CE Graves J Segal BM . Biological aging in multiple sclerosis. Mult Scler. (2023) 29:1701–8. doi: 10.1177/13524585231204122 37877740 PMC10843499

[9] Lopez-Otin C Blasco MA Partridge L Serrano M Kroemer G . The hallmarks of aging. Cell. (2013) 153:1194–217. doi: 10.1016/j.cell.2013.05.039 23746838 PMC3836174

[10] Lopez-Otin C Blasco MA Partridge L Serrano M Kroemer G . Hallmarks of aging: An expanding universe. Cell. (2023) 186:243–78. doi: 10.1016/j.cell.2022.11.001 36599349

[11] Graves JS Krysko KM Hua LH Absinta M Franklin RJM Segal BM . Ageing and multiple sclerosis. Lancet Neurol. (2023) 22:66–77. doi: 10.1016/S1474-4422(22)00184-3 36216015

[12] Childs BG Durik M Baker DJ van Deursen JM . Cellular senescence in aging and age-related disease: from mechanisms to therapy. Nat Med. (2015) 21:1424–35. doi: 10.1038/nm.4000 26646499 PMC4748967

[13] Munoz-Espin D Serrano M . Cellular senescence: from physiology to pathology. Nat Rev Mol Cell Biol. (2014) 15:482–96. doi: 10.1038/nrm3823 24954210

[14] Demaria M Ohtani N Youssef SA Rodier F Toussaint W Mitchell JR . An essential role for senescent cells in optimal wound healing through secretion of PDGF-AA. Dev Cell. (2014) 31:722–33. doi: 10.1016/j.devcel.2014.11.012 25499914 PMC4349629

[15] Xu C Qiu Z Guo Q Huang Y Zhao Y Zhao R . The role of cellular senescence in cardiovascular disease. Cell Death Discov. (2025) 11:431. doi: 10.1038/s41420-025-02720-5 41053058 PMC12501035

[16] Schafer MJ White TA Iijima K Haak AJ Ligresti G Atkinson EJ . Cellular senescence mediates fibrotic pulmonary disease. Nat Commun. (2017) 8:14532. doi: 10.1038/ncomms14532 28230051 PMC5331226

[17] Yousef A Fang L Heidari M Huang A Kondraciuk P Yee KA . Alterations in mitochondria and cellular senescence in aged sEH null female kidneys. Geroscience. (2026) 48:2707–25. doi: 10.1007/s11357-025-01814-3 40715936 PMC12972460

[18] Bhat R Crowe EP Bitto A Moh M Katsetos CD Garcia FU . Astrocyte senescence as a component of Alzheimer's disease. PloS One. (2012) 7:e45069. doi: 10.1371/journal.pone.0045069 22984612 PMC3440417

[19] Hayflick L Moorhead PS . The serial cultivation of human diploid cell strains. Exp Cell Res. (1961) 25:585–621. doi: 10.1016/0014-4827(61)90192-6 13905658

[20] Harley CB Futcher AB Greider CW . Telomeres shorten during ageing of human fibroblasts. Nature. (1990) 345:458–60. doi: 10.1038/345458a0 2342578

[21] Chang E Harley CB . Telomere length and replicative aging in human vascular tissues. Proc Natl Acad Sci USA. (1995) 92:11190–4. doi: 10.1073/pnas.92.24.11190 7479963 PMC40597

[22] Moyzis RK Buckingham JM Cram LS Dani M Deaven LL Jones MD . A highly conserved repetitive DNA sequence, (TTAGGG)n, present at the telomeres of human chromosomes. Proc Natl Acad Sci USA. (1988) 85:6622–6. doi: 10.1073/pnas.85.18.6622 3413114 PMC282029

[23] Demanelis K Jasmine F Chen LS Chernoff M Tong L Delgado D . Determinants of telomere length across human tissues. Science. (2020) 369:eaaz6876. doi: 10.1126/science.aaz6876 32913074 PMC8108546

[24] Armanios M Alder JK Parry EM Karim B Strong MA Greider CW . Short telomeres are sufficient to cause the degenerative defects associated with aging. Am J Hum Genet. (2009) 85:823–32. doi: 10.1016/j.ajhg.2009.10.028 19944403 PMC2790562

[25] Lin J Epel E Blackburn E . Telomeres and lifestyle factors: roles in cellular aging. Mutat Res. (2012) 730:85–9. doi: 10.1016/j.mrfmmm.2011.08.003 21878343

[26] Pepke ML Hansen SB Limborg MT . The ageing holobiont: crosstalk between telomere dynamics, oxidative stress and the gut microbiome. Biol Rev Camb Philos Soc. (2026) 101:1877–903. doi: 10.1002/brv.70152 41761700 PMC13326800

[27] Fumagalli M Rossiello F Clerici M Barozzi S Cittaro D Kaplunov JM . Telomeric DNA damage is irreparable and causes persistent DNA-damage-response activation. Nat Cell Biol. (2012) 14:355–65. doi: 10.1038/ncb2466 22426077 PMC3717580

[28] Zhu M-J Wang X Shi L Liang L-Y Wang Y . Senescence, oxidative stress and mitochondria dysfunction. Med Res Innov. (2018) 21:24. doi: 10.15761/MRI.1000149

[29] Yousef A Sosnowski DK Fang L Legaspi RJ Korodimas J Lee A . Cardioprotective response and senescence in aged sEH null female mice exposed to LPS. Am J Physiol Heart Circ Physiol. (2024) 326:H1366–85. doi: 10.1152/ajpheart.00706.2023 38578240

[30] Mohamad Kamal NS Safuan S Shamsuddin S Foroozandeh P . Aging of the cells: Insight into cellular senescence and detection Methods. Eur J Cell Biol. (2020) 99:151108. doi: 10.1016/j.ejcb.2020.151108 32800277

[31] Campisi J d'Adda di Fagagna F . Cellular senescence: when bad things happen to good cells. Nat Rev Mol Cell Biol. (2007) 8:729–40. doi: 10.1038/nrm2233 17667954

[32] Jiang X Stockwell BR Conrad M . Ferroptosis: mechanisms, biology and role in disease. Nat Rev Mol Cell Biol. (2021) 22:266–82. doi: 10.1038/s41580-020-00324-8 33495651 PMC8142022

[33] McKenzie BA Dixit VM Power C . Fiery cell death: Pyroptosis in the central nervous system. Trends Neurosci. (2020) 43:55–73. doi: 10.1016/j.tins.2019.11.005 31843293

[34] Shi J Gao W Shao F . Pyroptosis: Gasdermin-mediated programmed necrotic cell death. Trends Biochem Sci. (2017) 42:245–54. doi: 10.1016/j.tibs.2016.10.004 27932073

[35] Vandenabeele P Galluzzi L Vanden Berghe T Kroemer G . Molecular mechanisms of necroptosis: an ordered cellular explosion. Nat Rev Mol Cell Biol. (2010) 11:700–14. doi: 10.1038/nrm2970 20823910

[36] Tower J . Programmed cell death in aging. Ageing Res Rev. (2015) 23:90–100. doi: 10.1016/j.arr.2015.04.002 25862945 PMC4480161

[37] Papadopoulos D Magliozzi R Bandiera S Cimignolo I Barusolo E Probert L . Accelerated cellular senescence in progressive multiple sclerosis: A histopathological study. Ann Neurol. (2025) 97:1074–87. doi: 10.1002/ana.27195 39891488 PMC12081997

[38] Papadopoulos D Magliozzi R Mitsikostas DD Gorgoulis VG Nicholas RS . Aging, cellular senescence, and progressive multiple sclerosis. Front Cell Neurosci. (2020) 14:178. doi: 10.3389/fncel.2020.00178 32694983 PMC7338849

[39] Mijit M Caracciolo V Melillo A Amicarelli F Giordano A . Role of p53 in the regulation of cellular senescence. Biomolecules. (2020) 10:420. doi: 10.3390/biom10030420 32182711 PMC7175209

[40] Brooks CL Gu W . Ubiquitination, phosphorylation and acetylation: the molecular basis for p53 regulation. Curr Opin Cell Biol. (2003) 15:164–71. doi: 10.1016/s0955-0674(03)00003-6 12648672

[41] Shay JW Pereira-Smith OM Wright WE . A role for both RB and p53 in the regulation of human cellular senescence. Exp Cell Res. (1991) 196:33–9. doi: 10.1016/0014-4827(91)90453-2 1652450

[42] Mansilla SF de la Vega MB Calzetta NL Siri SO Gottifredi V . CDK-independent and PCNA-dependent functions of p21 in DNA replication. Genes (Basel). (2020) 11:593. doi: 10.3390/genes11060593 32481484 PMC7349641

[43] Rayess H Wang MB Srivatsan ES . Cellular senescence and tumor suppressor gene p16. Int J Cancer. (2012) 130:1715–25. doi: 10.1002/ijc.27316 22025288 PMC3288293

[44] Herranz N Gil J . Mechanisms and functions of cellular senescence. J Clin Invest. (2018) 128:1238–46. doi: 10.1172/JCI95148 29608137 PMC5873888

[45] Krishna DR Sperker B Fritz P Klotz U . Does pH 6 β-galactosidase activity indicate cell senescence? Mech Ageing Dev. (1999) 109:113–23. doi: 10.1016/s0047-6374(99)00031-7 10515661

[46] Dimri GP Lee X Basile G Acosta M Scott G Roskelley C . A biomarker that identifies senescent human cells in culture and in aging skin *in vivo*. Proc Natl Acad Sci. (1995) 92:9363–7. doi: 10.1073/pnas.92.20.9363 7568133 PMC40985

[47] Geng YQ Guan JT Xu XH Fu YC . Senescence-associated beta-galactosidase activity expression in aging hippocampal neurons. Biochem Biophys Res Commun. (2010) 396:866–9. doi: 10.1016/j.bbrc.2010.05.011 20457127

[48] Lopes-Paciencia S Saint-Germain E Rowell MC Ruiz AF Kalegari P Ferbeyre G . The senescence-associated secretory phenotype and its regulation. Cytokine. (2019) 117:15–22. doi: 10.1016/j.cyto.2019.01.013 30776684

[49] Tchkonia T Palmer AK Kirkland JL . New horizons: Novel approaches to enhance healthspan through targeting cellular senescence and related aging mechanisms. J Clin Endocrinol Metab. (2021) 106:e1481–7. doi: 10.1210/clinem/dgaa728 33155651 PMC7947756

[50] Lau L Porciuncula A Yu A Iwakura Y David G . Uncoupling the senescence-associated secretory phenotype from cell cycle exit via interleukin-1 inactivation unveils its protumorigenic role. Mol Cell Biol. (2019) 39:e00586–18. doi: 10.1128/MCB.00586-18 30988157 PMC6549465

[51] Chinta SJ Woods G Demaria M Rane A Zou Y McQuade A . Cellular senescence is induced by the environmental neurotoxin paraquat and contributes to neuropathology linked to Parkinson's disease. Cell Rep. (2018) 22:930–40. doi: 10.1016/j.celrep.2017.12.092 29386135 PMC5806534

[52] Lye JJ Latorre E Lee BP Bandinelli S Holley JE Gutowski NJ . Astrocyte senescence may drive alterations in GFAPalpha, CDKN2A p14(ARF), and TAU3 transcript expression and contribute to cognitive decline. Geroscience. (2019) 41:561–73. doi: 10.1007/s11357-019-00100-3 31654269 PMC6885035

[53] Wang H Pollock NM Miranzadeh Mahabadi H Patel J Moussa EW Fernandes JP . Brain and neural cell type proteomics reveal extracellular matrix proteins enriched in progressive multiple sclerosis. Nat Commun. (2026) 17:1368. doi: 10.1038/s41467-025-68118-0 41501060 PMC12877194

[54] Proescholdt MA Jacobson S Tresser N Oldfield EH Merrill MJ . Vascular endothelial growth factor is expressed in multiple sclerosis plaques and can induce inflammatory lesions in experimental allergic encephalomyelitis rats. J Neuropathol Exp Neurol. (2002) 61:914–25. doi: 10.1093/jnen/61.10.914 12387457

[55] Amstad A Coray M Frick C Barro C Oechtering J Amann M . Growth differentiation factor 15 is increased in stable MS. Neurol Neuroimmunol Neuroinflamm. (2020) 7:e675. doi: 10.1212/NXI.0000000000000675 32024796 PMC7051204

[56] Grist JJ Marro BS Skinner DD Syage AR Worne C Doty DJ . Induced CNS expression of CXCL1 augments neurologic disease in a murine model of multiple sclerosis via enhanced neutrophil recruitment. Eur J Immunol. (2018) 48:1199–210. doi: 10.1002/eji.201747442 29697856 PMC6033633

[57] Lassmann H van Horssen J Mahad D . Progressive multiple sclerosis: pathology and pathogenesis. Nat Rev Neurol. (2012) 8:647–56. doi: 10.1038/nrneurol.2012.168 23007702

[58] Lucchinetti CF Brueck W Rodriguez M Lassmann H . Multiple sclerosis: lessons from neuropathology. Semin Neurol. (1998) 18:337–49. doi: 10.1055/s-2008-1040885 9817538

[59] Elliott C Wolinsky JS Hauser SL Kappos L Barkhof F Bernasconi C . Slowly expanding/evolving lesions as a magnetic resonance imaging marker of chronic active multiple sclerosis lesions. Mult Scler. (2019) 25:1915–25. doi: 10.1177/1352458518814117 30566027 PMC6876256

[60] Frischer JM Weigand SD Guo Y Kale N Parisi JE Pirko I . Clinical and pathological insights into the dynamic nature of the white matter multiple sclerosis plaque. Ann Neurol. (2015) 78:710–21. doi: 10.1002/ana.24497 26239536 PMC4623970

[61] Jackle K Zeis T Schaeren-Wiemers N Junker A van der Meer F Kramann N . Molecular signature of slowly expanding lesions in progressive multiple sclerosis. Brain. (2020) 143:2073–88. doi: 10.1093/brain/awaa158 32577755

[62] Yong HYF Yong VW . Mechanism-based criteria to improve therapeutic outcomes in progressive multiple sclerosis. Nat Rev Neurol. (2022) 18:40–55. doi: 10.1038/s41582-021-00581-x 34732831

[63] Preziosa P Pagani E Meani A Moiola L Rodegher M Filippi M . Slowly expanding lesions predict 9-year multiple sclerosis disease progression. Neurol Neuroimmunol Neuroinflamm. (2022) 9:e1139. doi: 10.1212/NXI.0000000000001139 35105685 PMC8808355

[64] Giovannoni G Popescu V Wuerfel J Hellwig K Iacobaeus E Jensen MB . Smouldering multiple sclerosis: the 'real MS'. Ther Adv Neurol Disord. (2022) 15:17562864211066751. doi: 10.1177/17562864211066751 35096143 PMC8793117

[65] Leyane TS Jere SW Houreld NN . Oxidative stress in ageing and chronic degenerative pathologies: molecular mechanisms involved in counteracting oxidative stress and chronic inflammation. Int J Mol Sci. (2022) 23:7273. doi: 10.3390/ijms23137273 35806275 PMC9266760

[66] Absinta M Maric D Gharagozloo M Garton T Smith MD Jin J . A lymphocyte-microglia-astrocyte axis in chronic active multiple sclerosis. Nature. (2021) 597:709–14. doi: 10.1038/s41586-021-03892-7 34497421 PMC8719282

[67] Nicaise AM Wagstaff LJ Willis CM Paisie C Chandok H Robson P . Cellular senescence in progenitor cells contributes to diminished remyelination potential in progressive multiple sclerosis. Proc Natl Acad Sci USA. (2019) 116:9030–9. doi: 10.1073/pnas.1818348116 30910981 PMC6500153

[68] Safari-Alighiarloo N Rezaei-Tavirani M Taghizadeh M Tabatabaei SM Namaki S . Network-based analysis of differentially expressed genes in cerebrospinal fluid (CSF) and blood reveals new candidate genes for multiple sclerosis. PeerJ. (2016) 4:e2775. doi: 10.7717/peerj.2775 28028462 PMC5183126

[69] Daviaud N Chen E Edwards T Sadiq SA . Cerebral organoids in primary progressive multiple sclerosis reveal stem cell and oligodendrocyte differentiation defect. Biol Open. (2023) 12:bio059845. doi: 10.1242/bio.059845 36744877 PMC10040243

[70] Wilkins JM Gakh O Kabiraj P McCarthy CB Tobin WO Howe CL . Signatures of cell stress and altered bioenergetics in skin fibroblasts from patients with multiple sclerosis. Aging (Albany NY). (2020) 12:15134–56. doi: 10.18632/aging.103612 32640422 PMC7425440

[71] Park B Nicaise AM Tsitsipatis D Pirvan L Zucha D Munteanu A . Integrated omics reveals disease-associated radial glia-like cells with epigenetically dysregulated interferon response in multiple sclerosis. Neuron. (2025) 113:4158–77:e10. doi: 10.1016/j.neuron.2025.09.022 41075785 PMC12834227

[72] Fagiani F Pedrini E Martire MS Gastoldi G Vanden Bulcke C Lin JP . Spatially-restricted inflammation-induced senescent-like glia in multiple sclerosis and patient-derived organoids. Nat Commun. (2025) 16:8477. doi: 10.1038/s41467-025-63371-9 41006208 PMC12475462

[73] Itorralba J Brand-Arzamendi K Saab G Muccilli A Schneider R . Intrathecal interleukin-6 levels are associated with progressive disease and clinical severity in multiple sclerosis. BMC Neurol. (2025) 25:136. doi: 10.1186/s12883-025-04145-0 40175894 PMC11963510

[74] Gebka-Kepinska B Adamczyk B Gebka D Czuba Z Szczygiel J Adamczyk-Sowa M . Cytokine profiling in cerebrospinal fluid of patients with newly diagnosed relapsing-remitting multiple sclerosis (RRMS): associations between inflammatory biomarkers and disease activity. Int J Mol Sci. (2024) 25:7399. doi: 10.3390/ijms25137399 39000506 PMC11242697

[75] Donninelli G Studer V Brambilla L Zecca C Peluso D Laroni A . Immune soluble factors in the cerebrospinal fluid of progressive multiple sclerosis patients segregate into two groups. Front Immunol. (2021) 12:633167. doi: 10.3389/fimmu.2021.633167 33777018 PMC7988186

[76] Swardfager W Lanctot K Rothenburg L Wong A Cappell J Herrmann N . A meta-analysis of cytokines in Alzheimer's disease. Biol Psychiatry. (2010) 68:930–41. doi: 10.1016/j.biopsych.2010.06.012 20692646

[77] Chen X Hu Y Cao Z Liu Q Cheng Y . Cerebrospinal fluid inflammatory cytokine aberrations in Alzheimer's disease, Parkinson's disease and amyotrophic lateral sclerosis: a systematic review and meta-analysis. Front Immunol. (2018) 9:2122. doi: 10.3389/fimmu.2018.02122 30283455 PMC6156158

[78] Fu J Chen S Liu J Yang J Ou R Zhang L . Serum inflammatory cytokines levels and the correlation analyses in Parkinson's disease. Front Cell Dev Biol. (2023) 11:1104393. doi: 10.3389/fcell.2023.1104393 36875766 PMC9978777

[79] Banarjee R Basisty N . Plasma senescence associated secretory proteins: a new link to mild cognitive impairment. J Nutr Health Aging. (2025) 29:100561. doi: 10.1016/j.jnha.2025.100561 40279862 PMC12180040

[80] Wang Y Kuca K You L Nepovimova E Heger Z Valko M . The role of cellular senescence in neurodegenerative diseases. Arch Toxicol. (2024) 98:2393–408. doi: 10.1007/s00204-024-03768-5 38744709 PMC11272704

[81] Hsiao EC Koniaris LG Zimmers-Koniaris T Sebald SM Huynh TV Lee SJ . Characterization of growth-differentiation factor 15, a transforming growth factor beta superfamily member induced following liver injury. Mol Cell Biol. (2000) 20:3742–51. doi: 10.1128/MCB.20.10.3742-3751.2000 10779363 PMC85678

[82] Desmedt S Desmedt V De Vos L Delanghe JR Speeckaert R Speeckaert MM . Growth differentiation factor 15: a novel biomarker with high clinical potential. Crit Rev Clin Lab Sci. (2019) 56:333–50. doi: 10.1080/10408363.2019.1615034 31076013

[83] Montero R Yubero D Villarroya J Henares D Jou C Rodriguez MA . GDF-15 is elevated in children with mitochondrial diseases and is induced by mitochondrial dysfunction. PloS One. (2016) 11:e0148709. doi: 10.1371/journal.pone.0148709 26867126 PMC4750949

[84] Fujita Y Taniguchi Y Shinkai S Tanaka M Ito M . Secreted growth differentiation factor 15 as a potential biomarker for mitochondrial dysfunctions in aging and age-related disorders. Geriatr Gerontol Int. (2016) 16:17–29. doi: 10.1111/ggi.12724 27018280

[85] Yatsuga S Fujita Y Ishii A Fukumoto Y Arahata H Kakuma T . Growth differentiation factor 15 as a useful biomarker for mitochondrial disorders. Ann Neurol. (2015) 78:814–23. doi: 10.1002/ana.24506 26463265 PMC5057301

[86] Baeva ME Tottenham I Koch M Camara-Lemarroy C . Biomarkers of disability worsening in inactive primary progressive multiple sclerosis. J Neuroimmunol. (2024) 387:578268. doi: 10.1016/j.jneuroim.2023.578268 38157653

[87] Nohara S Ishii A Yamamoto F Yanagiha K Moriyama T Tozaka N . GDF-15, a mitochondrial disease biomarker, is associated with the severity of multiple sclerosis. J Neurol Sci. (2019) 405:116429. doi: 10.1016/j.jns.2019.116429 31476622

[88] Eschborn M Pawlitzki M Wirth T Nelke C Pfeuffer S Schulte-Mecklenbeck A . Evaluation of age-dependent immune signatures in patients with multiple sclerosis. Neurol Neuroimmunol Neuroinflamm. (2021) 8:e1094. doi: 10.1212/NXI.0000000000001094 34667129 PMC8529419

[89] Fulop T Larbi A Pawelec G Khalil A Cohen AA Hirokawa K . Immunology of aging: the birth of inflammaging. Clin Rev Allergy Immunol. (2023) 64:109–22. doi: 10.1007/s12016-021-08899-6 34536213 PMC8449217

[90] Thakolwiboon S Mills EA Yang J Doty J Belkin MI Cho T . Immunosenescence and multiple sclerosis: inflammaging for prognosis and therapeutic consideration. Front Aging. (2023) 4:1234572. doi: 10.3389/fragi.2023.1234572 37900152 PMC10603254

[91] Wherry EJ . T cell exhaustion. Nat Immunol. (2011) 12:492–9. doi: 10.1038/ni.2035 21739672

[92] Wherry EJ Ha SJ Kaech SM Haining WN Sarkar S Kalia V . Molecular signature of CD8+ T cell exhaustion during chronic viral infection. Immunity. (2007) 27:670–84. doi: 10.1016/j.immuni.2007.09.006 17950003

[93] Salminen A . Activation of immunosuppressive network in the aging process. Ageing Res Rev. (2020) 57:100998. doi: 10.1016/j.arr.2019.100998 31838128

[94] Naylor K Li G Vallejo AN Lee WW Koetz K Bryl E . The influence of age on T cell generation and TCR diversity. J Immunol. (2005) 174:7446–52. doi: 10.4049/jimmunol.174.11.7446 15905594

[95] Weyand CM Brandes JC Schmidt D Fulbright JW Goronzy JJ . Functional properties of CD4+ CD28- T cells in the aging immune system. Mech Ageing Dev. (1998) 102:131–47. doi: 10.1016/s0047-6374(97)00161-9 9720647

[96] Callender LA Carroll EC Beal RWJ Chambers ES Nourshargh S Akbar AN . Human CD8(+) EMRA T cells display a senescence-associated secretory phenotype regulated by p38 MAPK. Aging Cell. (2018) 17:e12675. doi: 10.1111/acel.12675 29024417 PMC5770853

[97] Sospedra M Martin R . Immunology of multiple sclerosis. Annu Rev Immunol. (2005) 23:683–747. doi: 10.1146/annurev.immunol.23.021704.115707 15771584

[98] Thewissen M Linsen L Somers V Geusens P Raus J Stinissen P . Premature immunosenescence in rheumatoid arthritis and multiple sclerosis patients. Ann N Y Acad Sci. (2005) 1051:255–62. doi: 10.1196/annals.1361.066 16126966

[99] Luchtman DW Ellwardt E Larochelle C Zipp F . IL-17 and related cytokines involved in the pathology and immunotherapy of multiple sclerosis: current and future developments. Cytokine Growth Factor Rev. (2014) 25:403–13. doi: 10.1016/j.cytogfr.2014.07.013 25153998

[100] Huan J Culbertson N Spencer L Bartholomew R Burrows GG Chou YK . Decreased FOXP3 levels in multiple sclerosis patients. J Neurosci Res. (2005) 81:45–52. doi: 10.1002/jnr.20522 15952173

[101] Claes N Fraussen J Vanheusden M Hellings N Stinissen P Van Wijmeersch B . Age-associated B cells with proinflammatory characteristics are expanded in a proportion of multiple sclerosis patients. J Immunol. (2016) 197:4576–83. doi: 10.4049/jimmunol.1502448 27837111

[102] Haegert DG Hackenbroch JD Duszczyszyn D Fitz-Gerald L Zastepa E Mason H . Reduced thymic output and peripheral naive CD4 T-cell alterations in primary progressive multiple sclerosis (PPMS). J Neuroimmunol. (2011) 233:233–9. doi: 10.1016/j.jneuroim.2010.12.007 21272945

[103] Canto-Gomes J Da Silva-Ferreira S Silva CS Boleixa D Martins da Silva A Gonzalez-Suarez I . People with primary progressive multiple sclerosis have a lower number of central memory T cells and HLA-DR(+) Tregs. Cells. (2023) 12:439. doi: 10.3390/cells12030439 36766781 PMC9913799

[104] Arneth B . Activated CD4+ and CD8+ T cell proportions in multiple sclerosis patients. Inflammation. (2016) 39:2040–4. doi: 10.1007/s10753-016-0441-0 27596006

[105] Fraussen J Marquez S Takata K Beckers L Montes Diaz G Zografou C . Phenotypic and Ig repertoire analyses indicate a common origin of IgD(-)CD27(-) double negative B cells in healthy individuals and multiple sclerosis patients. J Immunol. (2019) 203:1650–4. doi: 10.4049/jimmunol.1801236 31391234 PMC6736705

[106] Tieck MP Vasilenko N Ruschil C Kowarik MC . Peripheral memory B cells in multiple sclerosis vs. double negative B cells in neuromyelitis optica spectrum disorder: disease driving B cell subsets during CNS inflammation. Front Cell Neurosci. (2024) 18:1337339. doi: 10.3389/fncel.2024.1337339 38385147 PMC10879280

[107] Bar-Or A Fawaz L Fan B Darlington PJ Rieger A Ghorayeb C . Abnormal B-cell cytokine responses a trigger of T-cell-mediated disease in MS? Ann Neurol. (2010) 67:452–61. doi: 10.1002/ana.21939 20437580

[108] Griffiths P Baraniak I Reeves M . The pathogenesis of human cytomegalovirus. J Pathol. (2015) 235:288–97. doi: 10.1002/path.4437 25205255

[109] Ascherio A Munger KL Lennette ET Spiegelman D Hernan MA Olek MJ . Epstein-Barr virus antibodies and risk of multiple sclerosis: a prospective study. JAMA. (2001) 286:3083–8. doi: 10.1001/jama.286.24.3083 11754673

[110] Farrell RA Antony D Wall GR Clark DA Fisniku L Swanton J . Humoral immune response to EBV in multiple sclerosis is associated with disease activity on MRI. Neurology. (2009) 73:32–8. doi: 10.1212/WNL.0b013e3181aa29fe 19458321 PMC2848585

[111] Bjornevik K Cortese M Healy BC Kuhle J Mina MJ Leng Y . Longitudinal analysis reveals high prevalence of Epstein-Barr virus associated with multiple sclerosis. Science. (2022) 375:296–301. doi: 10.1126/science.abj8222 35025605

[112] Lyu L Li Q Wang C . EBV latency programs: molecular and epigenetic regulation and its role in disease pathogenesis. J Med Virol. (2025) 97:e70501. doi: 10.1002/jmv.70501 40685972 PMC12277944

[113] Babcock GJ Hochberg D Thorley-Lawson AD . The expression pattern of Epstein-Barr virus latent genes *in vivo* is dependent upon the differentiation stage of the infected B cell. Immunity. (2000) 13:497–506. doi: 10.1016/s1074-7613(00)00049-2 11070168

[114] Pender MP Csurhes PA Burrows JM Burrows SR . Defective T-cell control of Epstein-Barr virus infection in multiple sclerosis. Clin Transl Immunol. (2017) 6:e126. doi: 10.1038/cti.2016.87 28197337 PMC5292561

[115] Jaquiery E Jilek S Schluep M Meylan P Lysandropoulos A Pantaleo G . Intrathecal immune responses to EBV in early MS. Eur J Immunol. (2010) 40:878–87. doi: 10.1002/eji.200939761 20017197

[116] Lossius A Johansen JN Vartdal F Robins H Jurate Saltyte B Holmoy T . High-throughput sequencing of TCR repertoires in multiple sclerosis reveals intrathecal enrichment of EBV-reactive CD8+ T cells. Eur J Immunol. (2014) 44:3439–52. doi: 10.1002/eji.201444662 25103993

[117] Angelini DF Serafini B Piras E Severa M Coccia EM Rosicarelli B . Increased CD8+ T cell response to Epstein-Barr virus lytic antigens in the active phase of multiple sclerosis. PloS Pathog. (2013) 9:e1003220. doi: 10.1371/journal.ppat.1003220 23592979 PMC3623710

[118] Weng NP . Telomere and adaptive immunity. Mech Ageing Dev. (2008) 129:60–6. doi: 10.1016/j.mad.2007.11.005 18199471 PMC2276146

[119] Bellon M Nicot C . Telomere dynamics in immune senescence and exhaustion triggered by chronic viral infection. Viruses. (2017) 9:289. doi: 10.3390/v9100289 28981470 PMC5691640

[120] van Baarle D Nanlohy NM Otto S Plunkett FJ Fletcher JM Akbar AN . Progressive telomere shortening of Epstein-Barr virus-specific memory T cells during HIV infection: contributor to exhaustion? J Infect Dis. (2008) 198:1353–7. doi: 10.1086/592170 18816191

[121] Muller L Di Benedetto S . Immunosenescence and cytomegalovirus: exploring their connection in the context of aging, health, and disease. Int J Mol Sci. (2024) 25:753. doi: 10.3390/ijms25020753 38255826 PMC10815036

[122] Lin Z Gao H Wang B Wang Y . Cytomegalovirus infection and its relationship with leukocyte telomere length: a cross-sectional study. Mediators Inflammation. (2021) 2021:6675353. doi: 10.1155/2021/6675353 33628118 PMC7899777

[123] van de Berg PJ Griffiths SJ Yong SL Macaulay R Bemelman FJ Jackson S . Cytomegalovirus infection reduces telomere length of the circulating T cell pool. J Immunol. (2010) 184:3417–23. doi: 10.4049/jimmunol.0903442 20176738

[124] Oost W Talma N Meilof JF Laman JD . Targeting senescence to delay progression of multiple sclerosis. J Mol Med (Berl). (2018) 96:1153–66. doi: 10.1007/s00109-018-1686-x 30229272 PMC6208951

[125] Desu H Balthazard R Daigneault A Da Cal S Klement W Yu J . Peripheral blood age-sensitive immune markers in multiple sclerosis: relation to sex, cytomegalovirus status, and treatment. EBioMedicine. (2025) 112:105559. doi: 10.1016/j.ebiom.2025.105559 39837012 PMC11788784

[126] Duarte LF Gatica S Castillo A Kalergis AM Bueno SM Riedel CA . Is there a role for herpes simplex virus type 1 in multiple sclerosis? Microbes Infect. (2023) 25:105084. doi: 10.1016/j.micinf.2022.105084 36586461

[127] Khanna KM Bonneau RH Kinchington PR Hendricks RL . Herpes simplex virus-specific memory CD8+ T cells are selectively activated and retained in latently infected sensory ganglia. Immunity. (2003) 18:593–603. doi: 10.1016/s1074-7613(03)00112-2 12753737 PMC2871305

[128] Gudnadottir M Helgadottir H Bjarnason O Jonsdottir K . Virus isolated from the brain of a patient with multiple sclerosis. Exp Neurol. (1964) 9:85–95. doi: 10.1016/0014-4886(64)90008-1 14126126

[129] Bergstrom T Andersen O Vahlne A . Isolation of herpes simplex virus type 1 during first attack of multiple sclerosis. Ann Neurol. (1989) 26:283–5. doi: 10.1002/ana.410260218 2549851

[130] Ferrante P Mancuso R Pagani E Guerini FR Calvo MG Saresella M . Molecular evidences for a role of HSV-1 in multiple sclerosis clinical acute attack. J Neurovirol. (2000) 6 Suppl 2:S109–14. 10871797

[131] Sanders VJ Waddell AE Felisan SL Li X Conrad AJ Tourtellotte WW . Herpes simplex virus in postmortem multiple sclerosis brain tissue. Arch Neurol. (1996) 53:125–33. doi: 10.1001/archneur.1996.00550020029012 8639061

[132] Duarte LF Villalobos V Farias MA Rangel-Ramirez MA Gonzalez-Madrid E Navarro AJ . Asymptomatic herpes simplex virus brain infection elicits cellular senescence phenotypes in the central nervous system of mice suffering multiple sclerosis-like disease. Commun Biol. (2024) 7:811. doi: 10.1038/s42003-024-06486-x 38965360 PMC11224417

[133] Kritsilis M S VR Koutsoudaki PN Evangelou K Gorgoulis VG Papadopoulos D . Ageing, cellular senescence and neurodegenerative disease. Int J Mol Sci. (2018) 19:2937. doi: 10.3390/ijms19102937 30261683 PMC6213570

[134] Maupin EA Adams KL . Cellular senescence in glial cells: implications for multiple sclerosis. J Neurochem. (2025) 169:e16301. doi: 10.1111/jnc.16301 39831743 PMC11745082

[135] Rutledge J Oh H Wyss-Coray T . Measuring biological age using omics data. Nat Rev Genet. (2022) 23:715–27. doi: 10.1038/s41576-022-00511-7 35715611 PMC10048602

[136] Scahill RI Frost C Jenkins R Whitwell JL Rossor MN Fox NC . A longitudinal study of brain volume changes in normal aging using serial registered magnetic resonance imaging. Arch Neurol. (2003) 60:989–94. doi: 10.1001/archneur.60.7.989 12873856

[137] Lycklama a Nijeholt GJ . Reduction of brain volume in MS. MRI and pathology findings. J Neurol Sci. (2005) 233:199–202. doi: 10.1016/j.jns.2005.03.016 15949505

[138] Giovannoni G Butzkueven H Dhib-Jalbut S Hobart J Kobelt G Pepper G . Brain health: time matters in multiple sclerosis. Mult Scler Relat Disord. (2016) 9 Suppl 1:S5–S48. doi: 10.1016/j.msard.2016.07.003 27640924

[139] Azevedo CJ Cen SY Jaberzadeh A Zheng L Hauser SL Pelletier D . Contribution of normal aging to brain atrophy in MS. Neurol Neuroimmunol Neuroinflamm. (2019) 6:e616. doi: 10.1212/NXI.0000000000000616 32330116 PMC6807662

[140] Vollmer T Signorovitch J Huynh L Galebach P Kelley C DiBernardo A . The natural history of brain volume loss among patients with multiple sclerosis: a systematic literature review and meta-analysis. J Neurol Sci. (2015) 357:8–18. doi: 10.1016/j.jns.2015.07.014 26238166

[141] Calabrese M Romualdi C Poretto V Favaretto A Morra A Rinaldi F . The changing clinical course of multiple sclerosis: a matter of gray matter. Ann Neurol. (2013) 74:76–83. doi: 10.1002/ana.23882 23494723

[142] Jacobsen C Hagemeier J Myhr KM Nyland H Lode K Bergsland N . Brain atrophy and disability progression in multiple sclerosis patients: a 10-year follow-up study. J Neurol Neurosurg Psychiatry. (2014) 85:1109–15. doi: 10.1136/jnnp-2013-306906 24554101

[143] Baker DJ Wijshake T Tchkonia T LeBrasseur NK Childs BG van de Sluis B . Clearance of p16Ink4a-positive senescent cells delays ageing-associated disorders. Nature. (2011) 479:232–6. doi: 10.1038/nature10600 22048312 PMC3468323

[144] Zhang X Pearsall VM Carver CM Atkinson EJ Clarkson BDS Grund EM . Rejuvenation of the aged brain immune cell landscape in mice through p16-positive senescent cell clearance. Nat Commun. (2022) 13:5671. doi: 10.1038/s41467-022-33226-8 36167854 PMC9515187

[145] Bitto A Sell C Crowe E Lorenzini A Malaguti M Hrelia S . Stress-induced senescence in human and rodent astrocytes. Exp Cell Res. (2010) 316:2961–8. doi: 10.1016/j.yexcr.2010.06.021 20620137

[146] Salminen A Ojala J Kaarniranta K Haapasalo A Hiltunen M Soininen H . Astrocytes in the aging brain express characteristics of senescence-associated secretory phenotype. Eur J Neurosci. (2011) 34:3–11. doi: 10.1111/j.1460-9568.2011.07738.x 21649759

[147] Schlett JS Mettang M Skaf A Schweizer P Errerd A Mulugeta EA . NF-kappaB is a critical mediator of post-mitotic senescence in oligodendrocytes and subsequent white matter loss. Mol Neurodegener. (2023) 18:24. doi: 10.1186/s13024-023-00616-5 37069623 PMC10108549

[148] Krysko KM Henry RG Cree BAC Lin J University of California SFMSET Caillier S . Telomere length is associated with disability progression in multiple sclerosis. Ann Neurol. (2019) 86:671–82. doi: 10.1002/ana.25592 31486104 PMC7135931

[149] Piehl F . Current and emerging disease-modulatory therapies and treatment targets for multiple sclerosis. J Intern Med. (2021) 289:771–91. doi: 10.1111/joim.13215 33258193 PMC8246813

[150] Montalban X Hauser SL Kappos L Arnold DL Bar-Or A Comi G . Ocrelizumab versus placebo in primary progressive multiple sclerosis. N Engl J Med. (2017) 376:209–20. doi: 10.1056/NEJMoa1606468 28002688

[151] Kappos L Bar-Or A Cree BAC Fox RJ Giovannoni G Gold R . Siponimod versus placebo in secondary progressive multiple sclerosis (EXPAND): a double-blind, randomised, phase 3 study. Lancet. (2018) 391:1263–73. doi: 10.1016/S0140-6736(18)30475-6 29576505

[152] Giovannoni G Airas L Bove R Cutter GR Czarnecki M Drulovic J . Efficacy and safety of ocrelizumab in primary progressive multiple sclerosis, including older patients and those with more advanced disease (ORATORIO-HAND): a multicentre, double-blind, randomised, placebo-controlled, phase 3b study. Lancet. (2026) 407:2195–207. doi: 10.1016/S0140-6736(26)00617-3 42208561

[153] Weideman AM Tapia-Maltos MA Johnson K Greenwood M Bielekova B . Meta-analysis of the age-dependent efficacy of multiple sclerosis treatments. Front Neurol. (2017) 8:577. doi: 10.3389/fneur.2017.00577 29176956 PMC5686062

[154] Signori A Schiavetti I Gallo F Sormani MP . Subgroups of multiple sclerosis patients with larger treatment benefits: a meta-analysis of randomized trials. Eur J Neurol. (2015) 22:960–6. doi: 10.1111/ene.12690 25786797

[155] Vollmer BL Wolf AB Sillau S Corboy JR Alvarez E . Evolution of disease modifying therapy benefits and risks: an argument for de-escalation as a treatment paradigm for patients with multiple sclerosis. Front Neurol. (2021) 12:799138. doi: 10.3389/fneur.2021.799138 35145470 PMC8821102

[156] Corboy JR Fox RJ Kister I Cutter GR Morgan CJ Seale R . Risk of new disease activity in patients with multiple sclerosis who continue or discontinue disease-modifying therapies (DISCOMS): a multicentre, randomised, single-blind, phase 4, non-inferiority trial. Lancet Neurol. (2023) 22:568–77. doi: 10.1016/S1474-4422(23)00154-0 37353277

[157] Coerver EME Fung WH de Beukelaar J Bouvy WH Canta LR Gerlach OHH . Discontinuation of first-line disease-modifying therapy in patients with stable multiple sclerosis: the DOT-MS randomized clinical trial. JAMA Neurol. (2025) 82:123–31. doi: 10.1001/jamaneurol.2024.4164 39652340 PMC11811793

[158] Fraussen J Claes N Van Wijmeersch B van Horssen J Stinissen P Hupperts R . B cells of multiple sclerosis patients induce autoreactive proinflammatory T cell responses. Clin Immunol. (2016) 173:124–32. doi: 10.1016/j.clim.2016.10.001 27717695

[159] Goronzy JJ Weyand CM . Understanding immunosenescence to improve responses to vaccines. Nat Immunol. (2013) 14:428–36. doi: 10.1038/ni.2588 23598398 PMC4183346

[160] LeMaoult J Szabo P Weksler ME . Effect of age on humoral immunity, selection of the B-cell repertoire and B-cell development. Immunol Rev. (1997) 160:115–26. doi: 10.1111/j.1600-065x.1997.tb01032.x 9476670

[161] Kramer J Bar-Or A Turner TJ Wiendl H . Bruton tyrosine kinase inhibitors for multiple sclerosis. Nat Rev Neurol. (2023) 19:289–304. doi: 10.1038/s41582-023-00800-7 37055617 PMC10100639

[162] Oh J Bar-Or A Montalban X Sormani MP Weber MS Reich DS . Bruton's tyrosine kinase inhibitors: a new class of multiple sclerosis therapeutics. Lancet Neurol. (2026) 25:673–88. doi: 10.1016/S1474-4422(26)00141-9 42309088

[163] Atkinson J Dokiburra A Groover H Godbout JP Segal BM Zhang Y . Targeting senescent microglia in progressive multiple sclerosis: a geroscience-informed approach. Front Immunol. (2025) 16:1681724. doi: 10.3389/fimmu.2025.1681724 41126823 PMC12537384

[164] Xu M Palmer AK Ding H Weivoda MM Pirtskhalava T White TA . Targeting senescent cells enhances adipogenesis and metabolic function in old age. Elife. (2015) 4:e12997. doi: 10.7554/eLife.12997 26687007 PMC4758946

[165] Roos CM Zhang B Palmer AK Ogrodnik MB Pirtskhalava T Thalji NM . Chronic senolytic treatment alleviates established vasomotor dysfunction in aged or atherosclerotic mice. Aging Cell. (2016) 15:973–7. doi: 10.1111/acel.12458 26864908 PMC5013022

[166] Farr JN Xu M Weivoda MM Monroe DG Fraser DG Onken JL . Targeting cellular senescence prevents age-related bone loss in mice. Nat Med. (2017) 23:1072–9. doi: 10.1038/nm.4385 28825716 PMC5657592

[167] Xu M Pirtskhalava T Farr JN Weigand BM Palmer AK Weivoda MM . Senolytics improve physical function and increase lifespan in old age. Nat Med. (2018) 24:1246–56. doi: 10.1038/s41591-018-0092-9 29988130 PMC6082705

[168] Bussian TJ Aziz A Meyer CF Swenson BL van Deursen JM Baker DJ . Clearance of senescent glial cells prevents tau-dependent pathology and cognitive decline. Nature. (2018) 562:578–82. doi: 10.1038/s41586-018-0543-y 30232451 PMC6206507

[169] Hickson LJ Langhi Prata LGP Bobart SA Evans TK Giorgadze N Hashmi SK . Senolytics decrease senescent cells in humans: preliminary report from a clinical trial of Dasatinib plus Quercetin in individuals with diabetic kidney disease. EBioMedicine. (2019) 47:446–56. doi: 10.1016/j.ebiom.2019.08.069 31542391 PMC6796530

[170] Justice JN Nambiar AM Tchkonia T LeBrasseur NK Pascual R Hashmi SK . Senolytics in idiopathic pulmonary fibrosis: results from a first-in-human, open-label, pilot study. EBioMedicine. (2019) 40:554–63. doi: 10.1016/j.ebiom.2018.12.052 30616998 PMC6412088

[171] Saliev T Singh PB . Senotherapeutics for brain aging management. Neurol Int. (2025) 17:204. doi: 10.3390/neurolint17120204 41441223 PMC12736323

[172] Boots AW Haenen GR Bast A . Health effects of quercetin: from antioxidant to nutraceutical. Eur J Pharmacol. (2008) 585:325–37. doi: 10.1016/j.ejphar.2008.03.008 18417116

[173] Lindauer M Hochhaus A . Dasatinib. Recent Results Cancer Res. (2010) 184:83–102. doi: 10.1007/978-3-642-01222-8_7 20072833

[174] Gonzales MM Garbarino VR Kautz TF Palavicini JP Lopez-Cruzan M Dehkordi SK . Senolytic therapy in mild Alzheimer's disease: a phase 1 feasibility trial. Nat Med. (2023) 29:2481–8. doi: 10.1038/s41591-023-02543-w 37679434 PMC10875739

[175] Manavi Z Melchor GS Bullard MR Gross PS Ray S Gaur P . Senescent cell reduction does not improve recovery in mice under experimental autoimmune encephalomyelitis (EAE) induced demyelination. J Neuroinflamm. (2025) 22:101. doi: 10.1186/s12974-025-03425-3 40197319 PMC11974124

[176] Lombardo ER Pijewski RS Lustig JT Dhari Z Lahiri A Papile LE . Senolytic treatment induces oligodendrocyte dysfunction and demyelination in the corpus callosum. Proc Natl Acad Sci USA. (2026) 123:e2524897123. doi: 10.1073/pnas.2524897123 41843680 PMC13012039

[177] Zhu Y Tchkonia T Fuhrmann-Stroissnigg H Dai HM Ling YY Stout MB . Identification of a novel senolytic agent, navitoclax, targeting the Bcl-2 family of anti-apoptotic factors. Aging Cell. (2016) 15:428–35. doi: 10.1111/acel.12445 26711051 PMC4854923

[178] Robbins PD Jurk D Khosla S Kirkland JL LeBrasseur NK Miller JD . Senolytic drugs: reducing senescent cell viability to extend health span. Annu Rev Pharmacol Toxicol. (2021) 61:779–803. doi: 10.1146/annurev-pharmtox-050120-105018 32997601 PMC7790861

[179] Millar CL Iloputaife I Baldyga K Kuo J Tchkonia T Kirkland JL . Rationale and design of STAMINA: Senolytics to alleviate mobility issues and neurological impairments in aging, a geroscience feasibility study. Transl Med Aging. (2023) 7:109–17. doi: 10.1016/j.tma.2023.10.004 40248131 PMC12005345

[180] Kim EC Kim JR . Senotherapeutics: emerging strategy for healthy aging and age-related disease. BMB Rep. (2019) 52:47–55. doi: 10.5483/BMBRep.2019.52.1.293 30526770 PMC6386227

[181] Moiseeva O Deschenes-Simard X St-Germain E Igelmann S Huot G Cadar AE . Metformin inhibits the senescence-associated secretory phenotype by interfering with IKK/NF-kappaB activation. Aging Cell. (2013) 12:489–98. doi: 10.1111/acel.12075 23521863

[182] Yang Y Lu X Liu N Ma S Zhang H Zhang Z . Metformin decelerates aging clock in male monkeys. Cell. (2024) 187:6358–78:e29. doi: 10.1016/j.cell.2024.08.021 39270656

[183] Branigan GL Torrandell-Haro G Vitali F Brinton RD Rodgers K . Age and sex differences on anti-hyperglycemic medication exposure and risk of newly diagnosed multiple sclerosis in propensity score matched type 2 diabetics. Heliyon. (2022) 8:e11196. doi: 10.1016/j.heliyon.2022.e11196 36325137 PMC9618986

[184] De Keersmaecker AV Van Doninck E Popescu V Willem L Cambron M Laureys G . A metformin add-on clinical study in multiple sclerosis to evaluate brain remyelination and neurodegeneration (MACSiMiSE-BRAIN): study protocol for a multi-center randomized placebo controlled clinical trial. Front Immunol. (2024) 15:1362629. doi: 10.3389/fimmu.2024.1362629 38680485 PMC11046490

[185] Laberge RM Sun Y Orjalo AV Patil CK Freund A Zhou L . MTOR regulates the pro-tumorigenic senescence-associated secretory phenotype by promoting IL1A translation. Nat Cell Biol. (2015) 17:1049–61. doi: 10.1038/ncb3195 26147250 PMC4691706

[186] Bagherpour B Salehi M Jafari R Bagheri A Kiani-Esfahani A Edalati M . Promising effect of rapamycin on multiple sclerosis. Mult Scler Relat Disord. (2018) 26:40–5. doi: 10.1016/j.msard.2018.08.009 30219744

[187] Li XL Zhang B Liu W Sun MJ Zhang YL Liu H . Rapamycin alleviates the symptoms of multiple sclerosis in experimental autoimmune encephalomyelitis (EAE) through mediating the TAM-TLRs-SOCS pathway. Front Neurol. (2020) 11:590884. doi: 10.3389/fneur.2020.590884 33329339 PMC7728797

[188] Harrison DE Strong R Sharp ZD Nelson JF Astle CM Flurkey K . Rapamycin fed late in life extends lifespan in genetically heterogeneous mice. Nature. (2009) 460:392–5. doi: 10.1038/nature08221 19587680 PMC2786175

[189] Zhang Y Bokov A Gelfond J Soto V Ikeno Y Hubbard G . Rapamycin extends life and health in C57BL/6 mice. J Gerontol A Biol Sci Med Sci. (2014) 69:119–30. doi: 10.1093/gerona/glt056 23682161 PMC4038246

[190] Pignolo RJ Passos JF Khosla S Tchkonia T Kirkland JL . Reducing senescent cell burden in aging and disease. Trends Mol Med. (2020) 26:630–8. doi: 10.1016/j.molmed.2020.03.005 32589933 PMC7857028

[191] Marrie RA Dobson R Baranzini SE Salvetti M Bailey C Bebo B . Toward a global research agenda for preventing multiple sclerosis. Mult Scler. (2026) 32:151–62. doi: 10.1177/13524585251398381 41454472 PMC12916883

[192] Guan JZ Guan WP Maeda T Guoqing X GuangZhi W Makino N . Patients with multiple sclerosis show increased oxidative stress markers and somatic telomere length shortening. Mol Cell Biochem. (2015) 400:183–7. doi: 10.1007/s11010-014-2274-1 25424527

[193] Guan JZ Guan WP Maeda T . Vitamin E administration erases an enhanced oxidation in multiple sclerosis. Can J Physiol Pharmacol. (2018) 96:1181–3. doi: 10.1139/cjpp-2018-0246 30092167

[194] Lopez-Armas GDC Ramos-Marquez ME Navarro-Meza M Macias-Islas MA Saldana-Cruz AM Zepeda-Moreno A . Leukocyte telomere length predicts severe disability in relapsing-remitting multiple sclerosis and correlates with mitochondrial DNA copy number. Int J Mol Sci. (2023) 24:916. doi: 10.3390/ijms24020916 36674427 PMC9862686

[195] Yang JH Miner AE Fair A Kinkel R Graves JS . Senescence marker p16INK4a expression in patients with multiple sclerosis. Mult Scler Relat Disord. (2024) 84:105498. doi: 10.1016/j.msard.2024.105498 38359693

[196] Rasche L Heiserich L Behrens JR Lenz K Pfuhl C Wakonig K . Analysis of lymphocytic DNA damage in early multiple sclerosis by automated Gamma-H2AX and 53BP1 foci detection: a case control study. PloS One. (2016) 11:e0147968. doi: 10.1371/journal.pone.0147968 26820970 PMC4731473

[197] Hug A Korporal M Schroder I Haas J Glatz K Storch-Hagenlocher B . Thymic export function and T cell homeostasis in patients with relapsing remitting multiple sclerosis. J Immunol. (2003) 171:432–7. doi: 10.4049/jimmunol.171.1.432 12817027

[198] Habib R Ocklenburg S Hoffjan S Haghikia A Epplen JT Arning L . Association between shorter leukocyte telomeres and multiple sclerosis. J Neuroimmunol. (2020) 341:577187. doi: 10.1016/j.jneuroim.2020.577187 32050150

[199] Hecker M Fitzner B Jager K Buhring J Schwartz M Hartmann A . Leukocyte telomere length in patients with multiple sclerosis and its association with clinical phenotypes. Mol Neurobiol. (2021) 58:2886–96. doi: 10.1007/s12035-021-02315-y 33547621 PMC8128833

[200] Piedrabuena MA Correale J Farez MF Rodriguez Murua S Martinez Canyazo C Fiol M . Telomere length as a biomarker in multiple sclerosis. Mult Scler. (2024) 30:1258–67. doi: 10.1177/13524585241273054 39246285

[201] Diomede F Rajan TS D'Aurora M Bramanti P Merciaro I Marchisio M . Stemness characteristics of periodontal ligament stem cells from donors and multiple sclerosis patients: A comparative study. Stem Cells Int. (2017) 2017:1606125. doi: 10.1155/2017/1606125 29387088 PMC5745749

[202] Redondo J Sarkar P Kemp K Virgo PF Pawade J Norton A . Reduced cellularity of bone marrow in multiple sclerosis with decreased MSC expansion potential and premature ageing *in vitro*. Mult Scler. (2018) 24:919–31. doi: 10.1177/1352458517711276 28548004 PMC6029147

[203] Ma Y Wang M Chen X Ruan W Yao J Lian X . Telomere length and multiple sclerosis: A Mendelian randomization study. Int J Neurosci. (2024) 134:229–33. doi: 10.1080/00207454.2022.2098737 35791675

